# Genome-Wide Diversity of Goats From Indonesia and the Philippines Reveals Local Ancestry With Different Levels of Cosmopolitan Admixture

**DOI:** 10.1093/gbe/evag177

**Published:** 2026-07-17

**Authors:** Ryo Masuko, Ayin, Maho Masaoka, Fuki Kawaguchi, Shinji Sasazaki, Muhammad I A Dagong, Sri R A Bugiwati, Joseph S Masangkay, Jiaqi Wu, Takahiro Yonezawa, Johannes A Lenstra, Hideyuki Mannen

**Affiliations:** Laboratory of Animal Breeding and Genetics, Graduates School of Agricultural Science, Kobe University, Kobe, Japan; Laboratory of Animal Breeding and Genetics, Graduates School of Agricultural Science, Kobe University, Kobe, Japan; Laboratory of Animal Breeding and Genetics, Graduates School of Agricultural Science, Kobe University, Kobe, Japan; Laboratory of Animal Breeding and Genetics, Graduates School of Agricultural Science, Kobe University, Kobe, Japan; Laboratory of Animal Breeding and Genetics, Graduates School of Agricultural Science, Kobe University, Kobe, Japan; Faculty of Animal Science, Hasanuddin University, Makassar, South Sulawesi 90245, Indonesia; Faculty of Animal Science, Hasanuddin University, Makassar, South Sulawesi 90245, Indonesia; College of Veterinary Medicine, University of the Philippines, Los Baños, Philippines; Graduate School of Integrated Sciences for Life, Hiroshima University, Higashi-Hiroshima, Japan; Department of Molecular Life Science, Tokai University School of Medicine, Isehara, Japan; Graduate School of Integrated Sciences for Life, Hiroshima University, Higashi-Hiroshima, Japan; Faculty of Veterinary Medicine, Utrecht University, Utrecht, Netherlands; Laboratory of Animal Breeding and Genetics, Graduates School of Agricultural Science, Kobe University, Kobe, Japan

**Keywords:** 50K SNP, goats, Island Southeast Asia, Capra hircus, Indian ocean

## Abstract

In order to investigate the genetic diversity, differentiation, gene flows, and propagation routes of Island Southeast Asian (ISEA) goats, we genotyped 50K genome-wide SNPs in 57 Philippine and 30 Indonesian Katjang goats. Correlations of the distance from the domestication center and the genetic diversities of 21 Asian populations were significantly negative. A relatively high diversity of the Philippine population (*H*_e_ = 0.363) against the distance from the domestication center suggests a history of admixture, notably without large effects on their Katjang phenotype. This was confirmed by supervised PCA (Principal Component Analysis), *f*_3_ coancestry and *f*_4_ admixture analyses, Treemix-inferred migrations and model-based clustering (Admixture program). The inferred differential admixture of ISEA goats agreed with previously reported fixation of mtDNA haplogroup B in Indonesia, but not in the Philippines, and with high frequencies of African or European Y-chromosomal haplogroups that were not observed in Mainland Southeast Asia. We propose that admixture of cosmopolitan European and African goats into ISEA was mediated by a unique feature of the domestic goat, the maritime transport of goats during the colonial period as a source of provisions. This was followed by more recent imports of popular breeds such as Boer, and other cosmopolitan breeds.

Significant statement This study revealed that Philippine goats have a higher diversity than in Indonesian goats, which suggests a higher level of exotic admixture in Philippine goats. This can be explained by genetic introgression of European as well as African goats and Island Southeast Asian goats. These findings suggest maritime gene flow across the Indian Ocean and more recent genetic influences from commercial Boer and other cosmopolitan breeds.

## Introduction

Since their domestication in the Fertile Crescent ∼11,000 years ago, goats (*Capra hircus*) have become widespread throughout the world (Zeder and Hesse 2000). Their small size and adaptability to the environment (Pereira and Amorim 2010; Zheng et al. 2020) facilitated their parallel migration with human populations.

Previous studies of goats using mitochondrial DNA (mtDNA) control region sequences revealed six maternal haplogroups (A, B, C, D, F, and G) (Luikart et al. 2001; Joshi et al. 2004; Naderi et al. 2007; Lin et al. 2013; Waki et al. 2015). Haplogroup A is the most widespread in the world, whereas, haplogroup B is found at high frequency in Southeast Asia (Luikart et al. 2001; Naderi et al. 2007, 2008; Zhao et al. 2014). In contrast, a clear geographic differentiation of Y-chromosomal haplotypes (Y1AA, Y1AB, Y1B, Y2A, and Y2B) within continents indicated bottlenecks and expansions during the migration of goat populations (VarGoats Consortium et al. 2022). Y1AB dominates in northern China, and Y1AA with Y2B is predominant in the south. Y1B, Y2A, and Y2B have been observed predominantly in Europe, Africa, and East Asia, respectively (Pereira et al. 2008, 2009; Waki et al. 2015; VarGoats Consortium et al. 2022). The development of genome-wide SNP Chip for goats (Tosser-Klopp et al. 2014) allowed to infer propagation routes for goats (Colli et al. 2018; Kumar et al. 2018; Berihulay et al. 2019; Deniskova et al. 2021; Somenzi et al. 2022; Le et al. 2024; Senczuk et al. 2024; Yonezawa et al. 2026).

The Island Southeast Asian (ISEA) countries (Indonesia and the Philippines) are known to harbor a wide variety of organisms. ISEA has a history of trade and Islamic missionary work by Muslim merchants from the 9th century and colonial rule by Spain in the Philippines and the Netherlands in Indonesia from the 16th century (Skowronek 1998; Ricklefs et al. 2010; Mbeki and van Rossum 2017).

Native goats in both the Philippines and Indonesia originate from the Katjang (or Kacang) goat, which is widely distributed across Southeast Asia (Bondoc 2005; Suyadi et al. 2022). In Indonesia, the Katjang is the most common native variety found throughout the archipelago (Pakpahan et al. 2023). However, since 1925 during the Dutch colonial rule local goats were crossbred with Etawa (Jamnapari) goats imported from India. Nowadays, Indonesia harbors numerous breeds, including crosses of the South African Boer meat goat and the native Katjang. Although native goats still comprise the majority of the population, the population of pure native Katjang goats in Indonesia faces pressure from Peranakan Etawa (Etawa × Katjang) and Boer hybrids (Pakpahan et al. 2023). In contrast, there are no distinct breeds within the Philippine Katjang population.

Maternal lineage analysis of goats in ISEA revealed a low frequency of haplogroup A in Indonesia (Mannen et al. 2020; Masuko et al. 2025). Furthermore, mtDNA haplogroups suggested two propagation routes of goats into ISEA through the Asian continent—from Taiwan and continental China and from the Malay Peninsula (Masuko et al. 2025). In addition, gene flow between ISEA and Europe or Africa was inferred by the distribution of mtDNA haplogroup B in the South Africa and the presence of the SRY haplotype Y2A in ISEA, which is predominant in Europe and Africa but absent in Mainland Southeast Asia (MSEA) (Masuko et al. 2025).

Here we report genome-wide SNP diversity of goats in ISEA. We generated 50K SNP profiles of goats in ISEA from seven islands and six regions and revealed the levels of genetic diversity and identified the original and exotic influence on the ISEA goats.

## Results

### Datasets

We genotyped goats from ISEA (Philippines: *n* = 75, Indonesia: *n* = 30), South Asia (Bangladesh: *n* = 34 and Bhutan: *n* = 5), and MSEA (Vietnam: *n* = 7, Laos: *n* = 5, Cambodia: *n* = 18, and Myanmar: n = 5) populations using the Illumina goat SNP 50K bead chip (53,347 SNPs). In addition to analyses at the country level, we assessed the genetic diversity of six Philippine subpopulations. Cambodian goats were divided into Cambodia-M and Cambodia-P populations because of obvious differences in morphology (Yonezawa et al. 2026) and the frequency of the mtDNA haplogroup B (Lin et al. 2013) (see details in the Materials and Methods section).

### Genetic Diversity

**Fig. 1.**
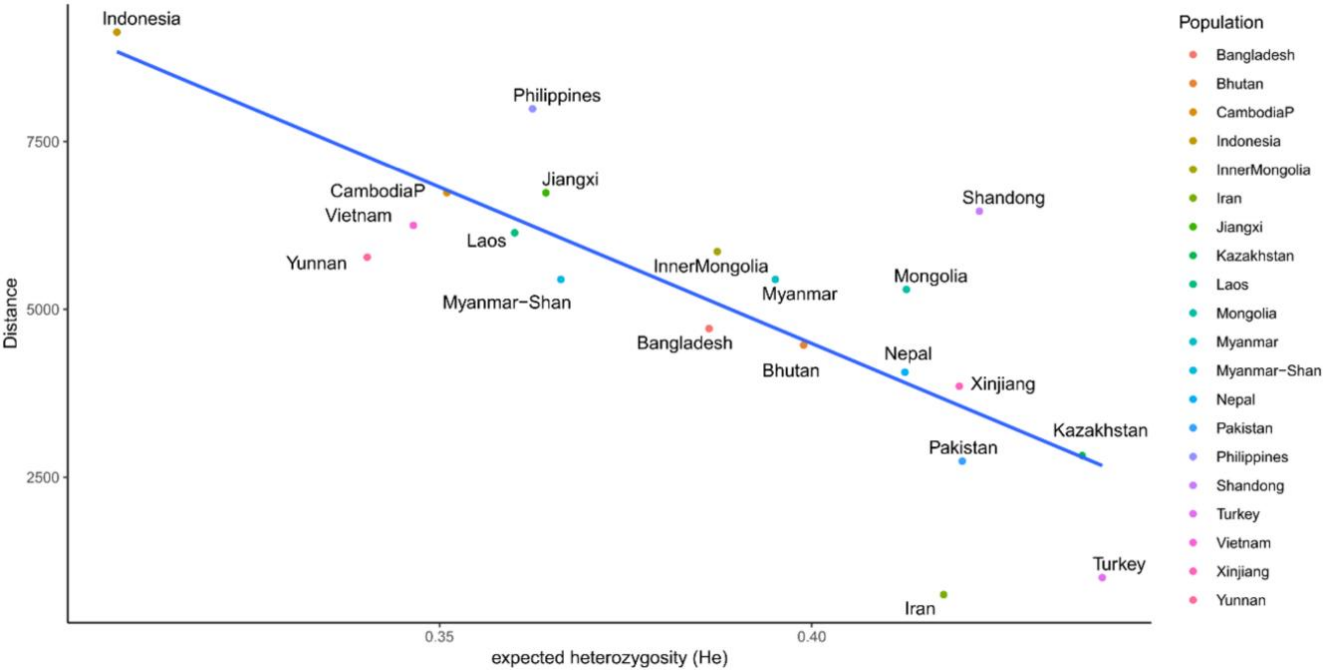
The correlation between expected heterozygosity of Asian goat population removing Cambodia-M and distance between their sampling locations and the domestication center. This plot was generated using RStudio 2023.09.0 + 463 “Desert Sunflower.”

Regional observed (*H*_o_) and expected (*H*_e_) heterozygosities (Table S1) indicated higher values for the Philippine goats (*H*_o_: 0.328; *H*_e_: 0.363) than those for the Indonesian goats (*H*_o_: 0.278; *H*_e_: 0.307). For the seven regions in ISEA, the *H*_o_ value ranged from 0.278 (Sulawesi) to 0.344 (Samar and Leyte), and the *H*_e_ value ranged from 0.307 (Sulawesi) to 0.355 (Samar and Leyte). Among Asian populations, the isolated Cambodia-M population had the lowest diversity (*H*_e_: 0.158). Without these outlier values, the *H*_e_ values correlated with the average distance from the sampling locations to the domestication center (*r* = −0.801, *P* = 1.278 × 10^−5^) (Figs. 1 and S1). The relatively high diversities in the Philippine and in Shandong populations suggested a mixed origin and were consistent with an *F*_ROH_ value based on runs of homozygosity (ROHs) of 500 bp. The *F*_ROH_ values showed 0.189 for the Philippine goats, which was lower than the value of 0.268 for the Indonesian goats. Within the six regions in the Philippines, the *F*_ROH_ values ranged from 0.150 (Samar and Leyte) to 0.289 (Mindanao). Nucleotide diversities (Pi) also confirmed a higher genetic diversity in the Philippines (3.38 × 10^−6^) than in Indonesia (2.71 × 10^−6^). SNP-based estimates of genetic diversity obtained in the present study were probably overestimated as a previous study showed that the 50K SNP array used here can bias estimates of genetic diversity (Benjelloun et al. 2019). However, this bias is unlikely to substantially affect comparisons among populations because all populations were compared using the same SNP dataset.

### Breed Relationships

**Fig. 2.**
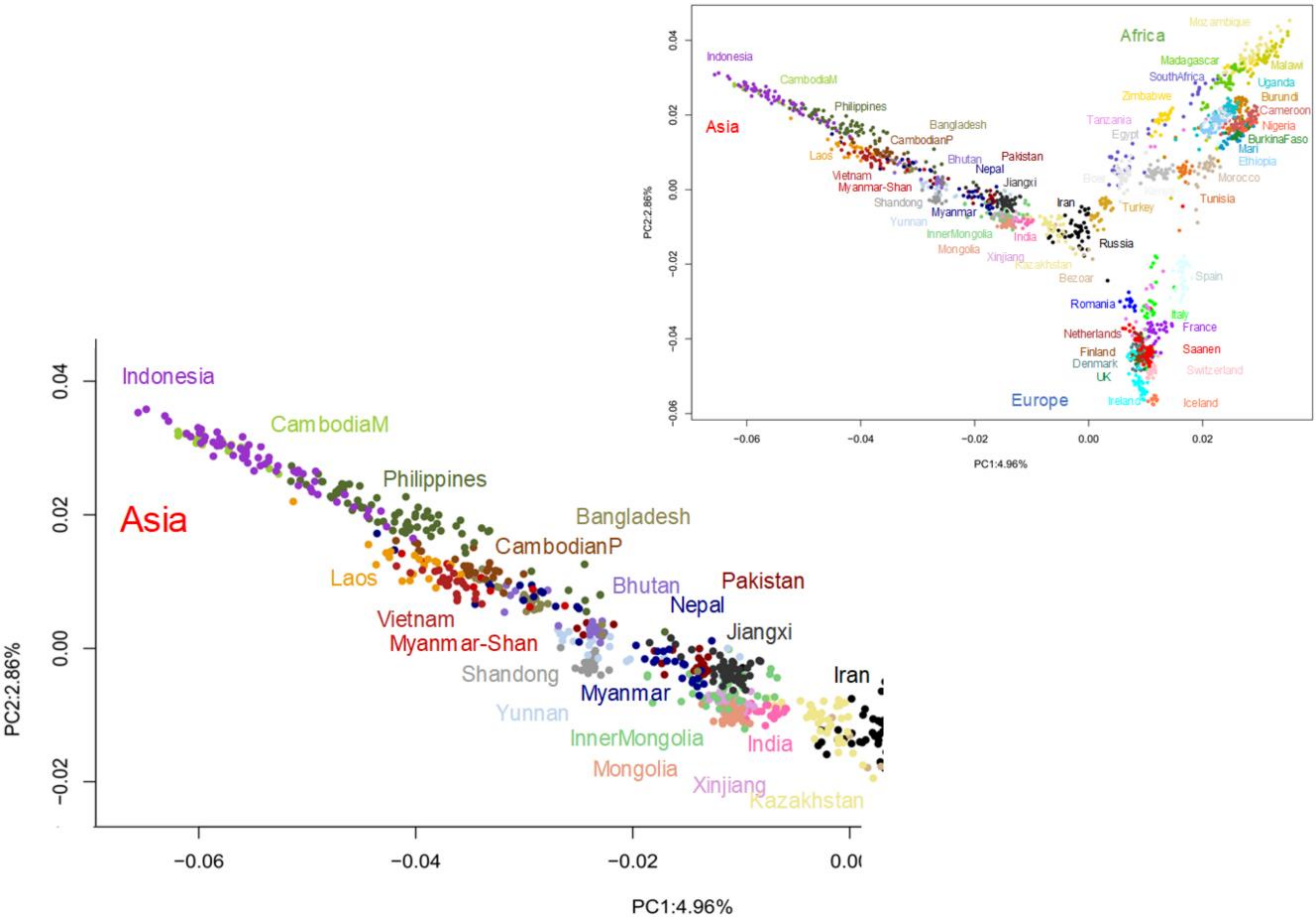
Supervised PCA of the Old World goats and the enlarged plot focused the Asian goats. These plots were generated by using RStudio 2023.09.0 + 463 “Desert Sunflower.”

We performed supervised PCA (Principal Component Analysis) (svPCA) to avoid the impact of high inbreeding in the Cambodia-M population, as described in our previous study (Yonezawa et al. 2026). In the unsupervised PCA, Cambodia-M goats had extreme positions (Fig. S2) but clustered with Southeast Asian (SEA) populations in the svPCA (Fig. 2). The PCA also made it possible to detect three clusters of European, Asian, and African breeds around the Middle East populations in the domestication center. PC1 (4.96%) distinguished Asia from Europe and Africa, and PC2 (2.86%) separated Europe from Asia and Africa.

ADMIXTURE model-based clustering from *K* = 1 to 40 showed the lowest cross-validation error at *K* = 33 as the optimal number of genomic components (Figs. S3 and S4). The plot of *K* = 3 confirmed the Asian, African, and European clusters, whereas at *K* ≥ 5, the ISEA populations were clearly differentiated from the other populations. We noted that Cambodia-M and Boer became inferred genomic components at *K* = 7 and *K* = 9, respectively, with clearly different contributions to the Philippine and Indonesian goats. For Cambodia-M, this agreed with the svPCA.

**Fig. 3.**
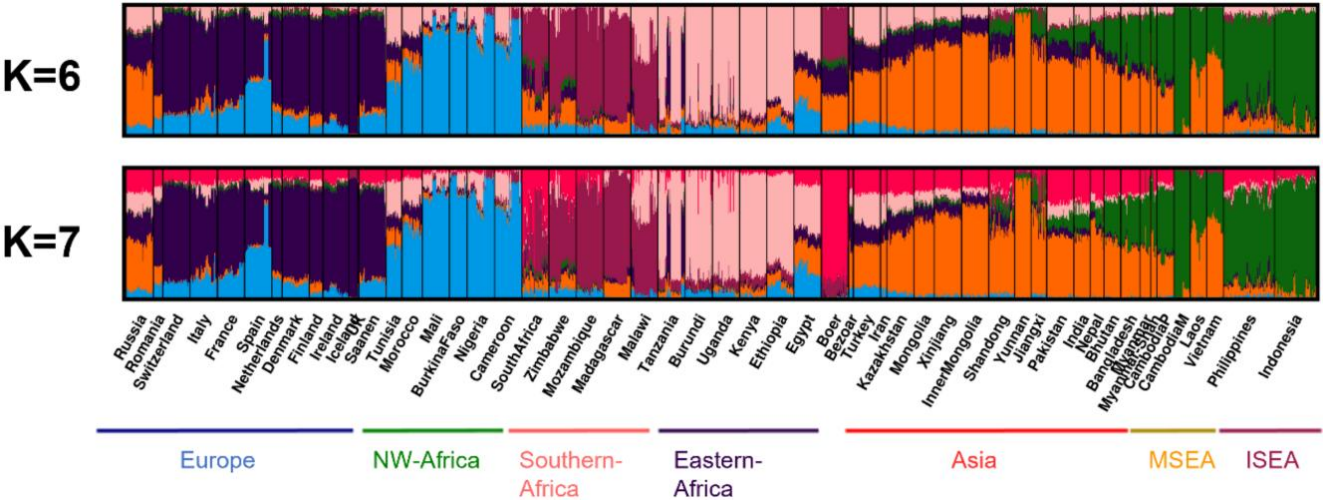
Reduced representation admixture analysis (RRAA) carried out with the ADMIXTURE program v.1.3 (*K* = 6 to 7). This figure was generated using CLUMPAK (https://clumpak.tau.ac.il/). NW-Africa, North/West-Africa; SEA, Southeast Asia; MSEA, Mainland Southeast Asia; ISEA, Island Southeast Asia.

In order to exclude the patterns that are distorted by the extremely low diversity of Cambodia-M (Lawson et al. 2018), we used the RRAA (reduced representation admixture analysis) resampling strategy (Yonezawa et al. 2026). Specifically, we averaged the genomic ancestry proportions across 100 independent runs, each involving a random subset of six Cambodia-M individuals. At *K* = 2 to 5, RRAA (Fig. S5) generated the same clusters as ADMIXTURE, but reducing the inbreeding bias showed an East-African cluster at *K* = 6 and a Boer-specific cluster at *K* = 7, instead of the Cambodia-M dominated cluster (Fig. 3).

**Fig. 4.**
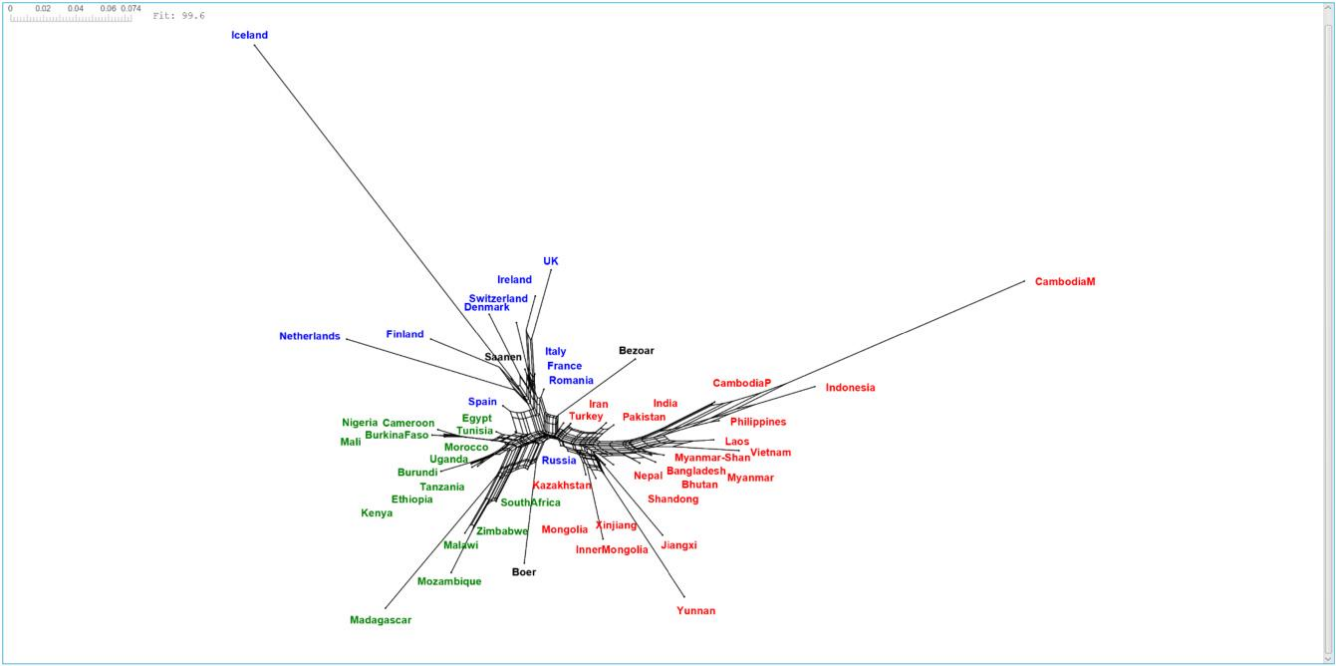
Neighbor-network of the Old World goats using SplitsTree App. The edge lengths are proportional to the pairwise *F*_ST_ distances between populations: Blue, green, and red colors showed Europe, Africa, and Asian populations, respectively.

*F*_ST_ genetic distances (Table S2) for goats in ISEA generally correlated with geographic distance. A neighbor-network based on the pairwise *F*_ST_ distance again separated Asia, Africa, and Europe (Fig. 4) and also clustered ISEA and MSEA. Long terminal branches for the Iceland, Madagascar, and Cambodia-M populations confirmed their genetic isolation reported previously (Colli et al. 2018; Yonezawa et al. 2026).

**Fig. 5.**
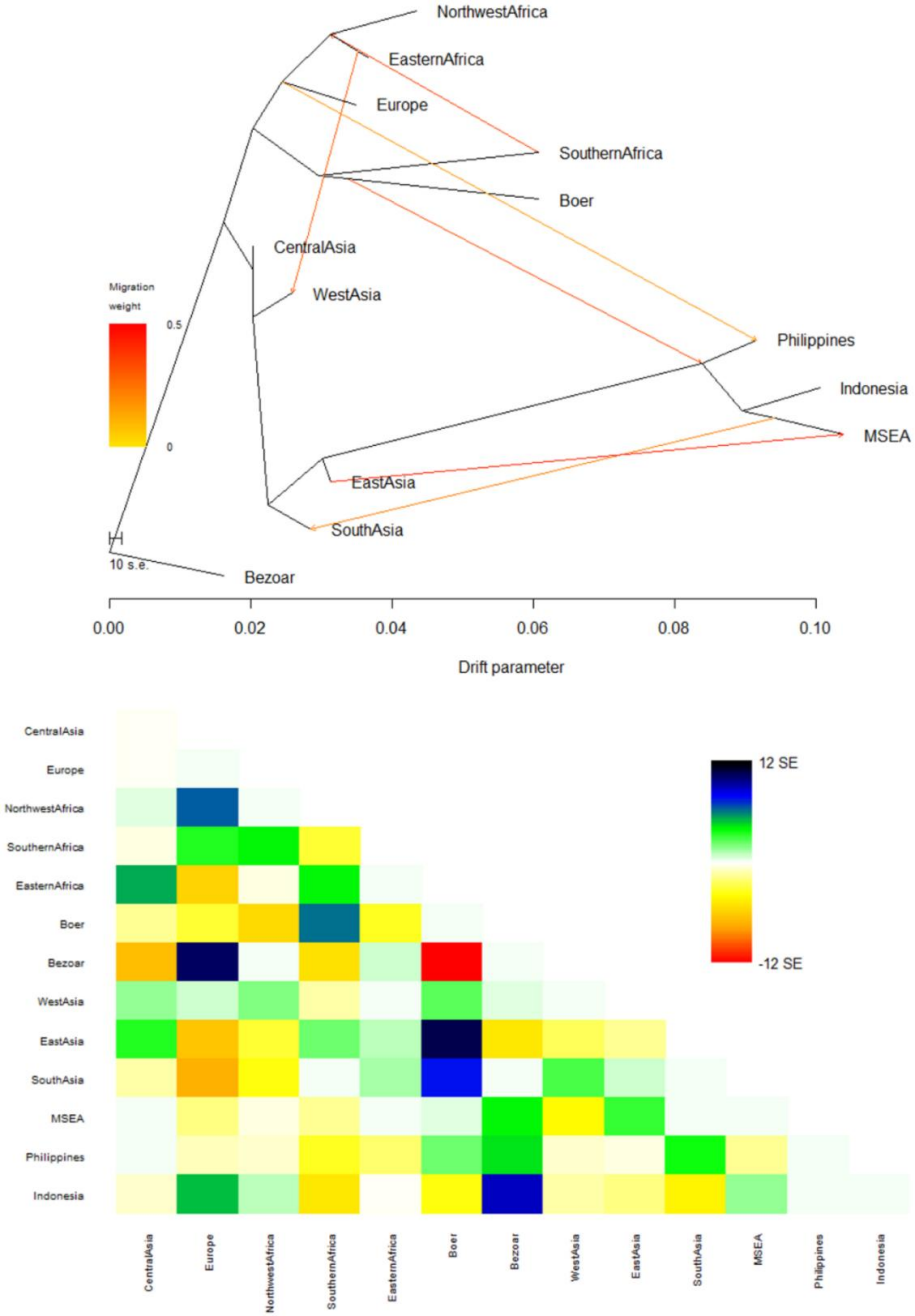
Regional Treemix graphs and residual plot at *m* = 6. The model was selected as the best model by the optM (lower). Bezoars were used as the root of the tree. Migration edges are colored in accordance with their migration weights. Scale bar indicates the drift parameter.

The same geographic clusters were generated by Treemix analyses of the gene flow (Figs. 5 and S6). Most patterns for 1 to 15 migrations showed migration edges from Boer to the Philippines goats. For *m* = 4 or higher, several migrations linked European populations with ISEA. However, Treemix may not identify all admixture events and also occasionally infer an incorrect direction of migration (Ciani et al. 2020; Lewald et al. 2021).

### Coancestries and Admixtures

**Fig. 6.**
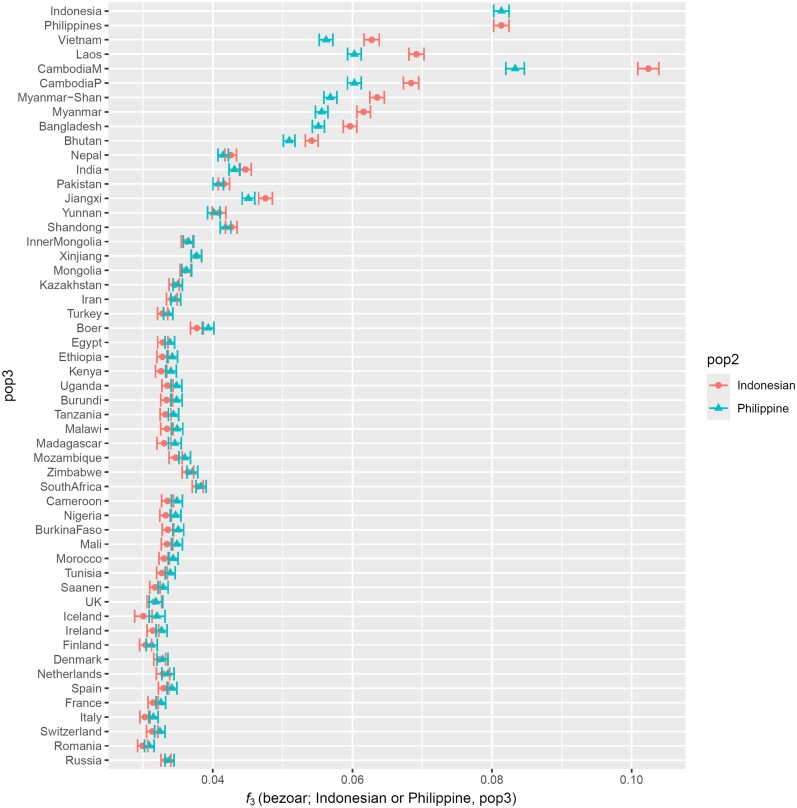
*f*_3_-Statistics between ISEA goats and other goats with bezoar as outgroup. This plot was generated using RStudio 2023.09.0 + 463 “Desert Sunflower.”

We calculated the outgroup coancestry *f*_3_ and the admixtures *f*_4_ statistics for the pairs of ISEA and other domesticated goat populations. The *f*_3_ statistic (bezoar; ISEA goats, other populations) estimated the genetic distance between the bezoar and the divergence of ISEA and the other populations and were generally inversely proportional to geographic distance with ISEA (Fig. 6). As expected, the higher values were found for Cambodia-M followed by the other Southeast-Asian population. Lower but significant coancestries were estimated for European and African populations. Interestingly, the South Africa and Boer populations had more coancestry with Philippines and Indonesia than other European or African populations. It was also remarkable that the Indonesian goats had more coancestry with the SEA populations than the Philippine goats, while the reverse was found for the European and African populations.

These trends were also reflected by admixture statistics (Table S3; Table 1 with selected values for *f*_4_ (bezoar, source (Africa/Europe/Asian populations); Vietnam as non-admixed control, target (Indonesia or Philippines)): Indonesian goats showed higher Asian admixtures than the Philippine goats but lower European or African admixtures. From the European and African populations, the highest *f*_4_ values were found for the South African and Boer introgressions in the Philippines. The different admixtures in Indonesian and Philippine goats agreed with the values of *f*_4_ (bezoar, source [Africa/Europe/Asian populations]; Indonesia, Philippines), which correlated the contrast between the two ISEA countries with the difference between the outgroup and source populations.

**Table 1:** Selected *f*_4_ values indicating Asian, European, and African influence on ISEA populations

*f*_4_ (Bezoar = outgroup, source; control = Vietnam, target = Indonesia/Philippines)			
for *z*-values with *f*_4_ > 0			
	Target	Indonesia	Philippines
Source-continent	Source		
Europe North	Denmark	2.06	3.02
Europe North	Finland	1.89	3.67
Europe North	Ireland	4.11	6.82
Europe North	Netherlands	3.04	4.47
Europe North	UK	4.73	5.51
Europe Central	France	3.71	6.39
Europe Central	Saanen	3.62	6.36
Europe Central	Switzerland	2.88	5.31
Europe South	Italy	2.61	5.45
Europe South	Romania	0.94	3.15
Europe South	Spain	3.19	5.85
Africa	Morocco	1.25	3.93
Africa	Nigeria	1.73	4.46
Africa	Cameroon	1.65	4.21
Africa	Ethiopia	0.54	3.33
Africa	Kenya	0.92	3.71
Africa	Burundi	1.06	3.77
Africa	Uganda	0.76	3.32
Africa South	Malawi	1.22	3.83
Africa South	Mozambique	1.59	3.77
Africa South	Zimbabwe	2.87	4.27
Africa South	South Africa	3.74	4.92
Africa South	Boer	3.14	6.00
Asia	India	5.99	3.41
Asia	Pakistan	3.61	2.31
Asia	Bhutan	10.98	5.97
Asia	Bangladesh	16.03	9.29
Asia Southeast	Myanmar	14.56	4.90
Asia Southeast	Cambodia-P	13.57	2.92
Asia Southeast	Cambodia-M	20.37	4.64

*f*_4_ (Bezoar = outgroup, source; Indonesia, Philippines)			
*z* < 0: more admixture in Indonesia; *z* > 0: more admixture in Philippines			
Source-continent	Source	*z* if |*z*|>3	
Europe North	Iceland	3.43	…
Europe North	Ireland	3.21	…
Europe Central	France	3.33	…
Europe Central	Saanen	3.38	…
Europe South	Italy	3.68	…
Europe South	Spain	3.55	…
Africa	BurkinaFaso	3.80	…
Africa	Burundi	3.68	…
Africa	Cameroon	3.58	…
Africa	Egypt	3.17	…
Africa	Ethiopia	4.05	…
Africa	Kenya	3.91	…
Africa	Mali	3.76	…
Africa	Morocco	3.78	…
Africa	Nigeria	3.81	…
Africa	Tanzania	3.38	…
Africa	Tunisia	3.47	…
Africa	Uganda	3.60	…
Africa South	Boer	3.96	…
Africa South	Mozambique	3.16	…
Africa South	Madagascar	3.30	…
Africa South	Malawi	3.63	…
Asia	Bhutan	−8.86	…
Asia	India	−4.50	…
Asia	Jiangxi	−5.90	…
Asia	Nepal	−3.14	…
Asia Southeast	Cambodia-M	−24.91	…
Asia Southeast	Cambodia-P	−17.54	…
Asia Southeast	Laos	−19.59	…
Asia Southeast	Myanmar	−15.92	…
Asia Southeast	Myanmar–Shan	−16.91	…
Asia Southeast	Vietnam	−13.82	…

The *f*_3_- and *f*_4_-inferred admixtures were confirmed by the genomic compositions of the ISEA populations estimated by the RRAA at *K* = 7 (Table S4): a substantial Southeast-Asian component that was higher in Indonesia than in the Philippines (86.6% vs 68.3%), and introgressions by African and European goats that were the highest in the Philippines (Boer 6.5% in Indonesia vs 13.5% in the Philippines; Europe 0.5% vs 3.7%; etc.). We further noted Southeast-Asian components in goats from South Africa (3.8%), Zimbabwe (3.3%), the Dutch (3.7%), and Danish (2.5%) landraces and in the English Bagot (2.6%) and Old Irish goats (3.0%).

## Discussion

This study focused on the ancestry of the Indonesian and Philippine goats, which in addition to their SEA roots involved different introgressions. In agreement with a previous study (Yonezawa et al. 2026), PCA, Admixture, RRAA, NeighborNet as well as Treemix linked both ISEA populations to the Indochinese MSEA goats. The low genetic diversity of Indonesian goats is explained by their long distance from the domestication center in Southwest Asia. However, this was not observed for the Philippine goats, which also occupied more basal positions in the svPCA and NeighborNet plots, suggesting a mixed origin that has decreased the original Southwest-Asian ancestry in the Philippines. The higher diversity of the Philippine goats agrees with the observation that the mtDNA haplogroup B is nearly fixed in Indonesia, but not in the Philippines (Masuko et al. 2025).

An obvious explanation is crossing of nearby Chinese goats into the Philippine population, but this is not supported by the low *f*_3_ coancestry between Philippine and Chinese goats and also would not explain the occurrence in the Philippine goats of the Mediterranean-African Y-chromosomal haplogroup Y2A. Intriguingly, Admixture- and RRAA-inferred genomic compositions as well as the *f*_3_ coancestry and *f*_3_ admixture indices suggest influence of Boer, African, and European goats, which is consistent with the Treemix-reconstructed migrations. These gene flows affected both ISEA populations, but had more influence on the Philippine populations (together accounting for 32% of their genome on the basis of RRAA *K* = 7 genomic components) than on the Indonesian population (13%). Notably, these values and all other estimates of admixture levels depend on the program algorithm (Dias-Alves et al. 2018), *K*-value, allele frequencies in the source populations that may differ from the present frequencies and other confounding factors difficult to assess. Therefore, these values should be considered as semi-quantitative relative estimates.

This cosmopolitan admixture in ISEA has left its traces in Y-chromosomal diversity. The Mediterranean-African haplogroup Y1AA is not present in Mainland SEA goats, but is carried by 17.9% of the Indonesian male goats and by 18.8% of the SEA goats, whereas 6.0% of the Philippine male goats carry the European haplogroup Y1B. It is also remarkable that both ISEA populations have absorbed this exotic influence without major effects on their Katjang phenotype.

**Fig. 7.**
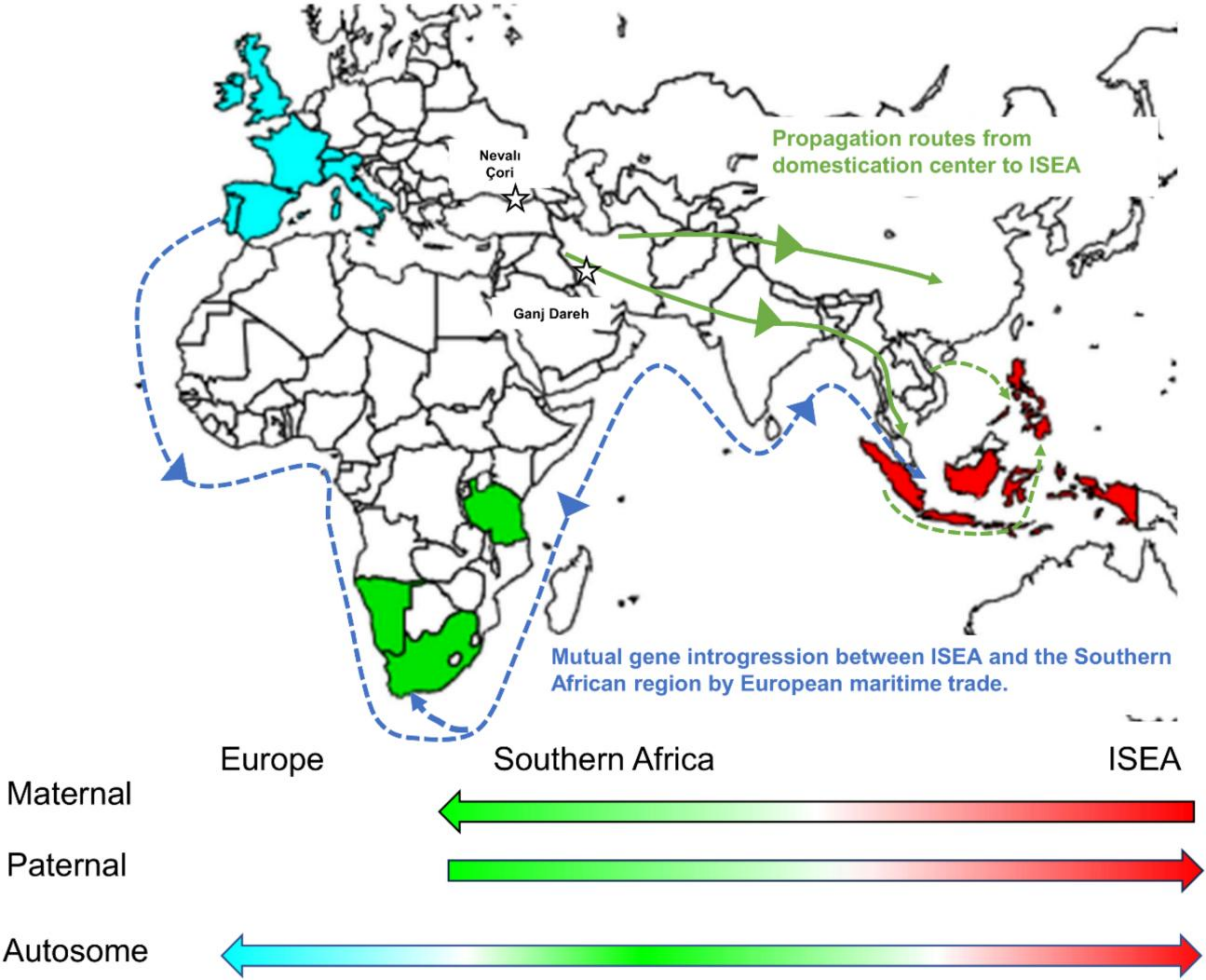
Assuming genetic influence from maritime trade during the Middle Ages, gene flow likely occurred through the Indian Ocean. Red, green, and blue colors show ISEA, the Southern African, and European regions, respectively. Green solid line shows the propagation routes from the domestication center to ISEA (Masuko et al. 2025; Yonezawa et al. 2026). Blue dot line shows the migration route based on historical voyage record and mutual gene flow between ISEA and the Southern African region. The map was generated using Inkscape 1.4 (86a8ad7, 2024-10-11) based on a free map website by 3kaku-K (https://www.freemap.jp/item/world/world1.html).

In order to explain our observations on the basis of human migrations and activities, we propose the following scenario:

1. The gene flow occurred in Southeast Asia no earlier than the Christian era and possibly no earlier than the Arabian invasion in the 14th century (Mason 1984). *F*_ST_ and *f*_3_ values showed a close relationship of Cambodia-M and Indonesian goats, suggesting that Cambodia-M and Katjang goats are closely related to the ancestors of the ISEA goats.
2. During the Age of Discovery from the 15th century, Portuguese and Dutch spice traders crossed the Indian Ocean (Chias and Abad 2012; Zuhdi 2018), whereas Spanish explorers arrived via the Pacific Ocean. Eventually, this resulted in the Dutch conquest of Indonesia and the Spanish conquest of the Philippines. According to historical records, chickens, sheep, and, most often, goats were transported aboard ships as a source of provisions (Lobo et al. 1971; Mason 1984; Porter et al. 2016; Casimiro and Borges 2023). These animals originated from Europe or from harbors along the African, Asian, or American coasts, which served as relay points. Because goat milk was used as a protein source, both female and male goats may have been transported. After the Spanish conquest, the Philippines became an important hub for trade with Asia (Skowronek 1998). In South Africa, the Cape Colony was established by the Dutch East India Company in the 17th century and used as a port of call for Oriental trade (Fourie et al. 2013). The maritime transport of goats during these trading trips has created several island populations (Weaver 2021) and may very well have catalyzed a gene flow of goats from several origins to ISEA as suggested by the present study (Figs. 3, 5 and 7; Table S4).

In other livestock species large-scale gene flow during the colonial period has been reported as well, such as for pigs brought to America (Crosby 2003), sheep (Kijas et al. 2012), and cattle (Ajmone-Marsan et al. 2010), but the widespread use of goats as provisions during maritime voyages is unique to this species.

3. Similar maritime transport of goats during return trips introduced Asian and African goats to England, where crossing with native English goats gave rise to the Anglo-Nubian breed. Another popular and major goat breed, the Boer, was created in South Africa by crossing Asian goats of the Jamnapari type with local as well as European imports (Casey and Van Niekerk 1988; Erasmus 2000; Malan 2000). Remarkably, minor inferred genomic components suggest tentative evidence for SEA ancestry in South African goats and also in the Dutch Landrace, the Danish Landrace, the English Bagot, and the Old Irish breed (Table S4). Although outside the scope of the present study, this may warrant further investigation.
4. During the last decades, several highly productive goat breeds have been imported, such as the Boer, Anglo-Nubian, and the Swiss dairy Alpine, Saanen, and Toggenburg (Porter et al. 2016; Widyas et al. 2021). Most of these goats are kept separately, but their import has also created an opportunity for crossing in Katjang populations. This probably accounts at least partially for the relatively large influx of Boer variants into the ISEA Katjang population. The Anglo-Nubian goats may have introduced the Y-chromosomal Y2A haplotype (VarGoats Consortium et al. 2022).

We conclude that the Indonesian and Philippine populations share an SEA origin, which especially for the Philippine goats has been eroded by successive admixtures of cosmopolitan goats. Phased datasets with a higher density of variants may provide accurate quantification and dating of different admixture events, which have remarkably preserved their local adaptation and identity of the ISEA Katjang goats.

## Materials and Methods

### **Ethics** Declarations

This study is reported according to ARRIVE guidelines (https://arriveguidelines.org). All experiments were conducted according to Kobe University Animal Experimentation Regulations, and all protocols were approved by the Institutional Animal Care and Use Committee of Kobe University and by the Association for the Promotion of Research Integrity (Approval Number: AP0000436777). All blood sample collections were approved by animal owners with signed informed consent.

### Sample Collection and Genotyping

DNA samples from 87 native goats in ISEA were obtained, including samples from the Philippines (*n* = 57) and Indonesia (*n* = 30) (Fig. S7). To compare the impact of gene flow from the Indian Ocean into ISEA with that from South and MSEA, where the mtDNA haplogroup B was primarily observed, we also newly obtained the DNA samples of 74 native goats from six countries in South Asia and MSEA, including Vietnam (*n* = 7), Laos (*n* = 5), Cambodia (*n* = 18), Myanmar (*n* = 5), Bangladesh (*n* = 34), and Bhutan (*n* = 5) (Fig. S8; Table S5). To avoid sampling-related individuals and hybrid individuals with international breeds, we first conducted interviews with farmers to exclude individuals with known parent–offspring or full–sibling relationships.

We also obtained previously published data from South Asia, MSEA, and ISEA for comparison (Yonezawa et al. 2026). Based on a previous study (Yonezawa et al. 2026), Cambodian goats were divided into Cambodia-M population (*n* = 11) found in a remote mountainous area east of the Mekong and Cambodia-P population (*n* = 7) found in the plain west of the Mekong, which showed obvious differences in morphology (Yonezawa et al. 2026) and in the frequency of the mtDNA haplogroup B (Lin et al. 2013). Similarly, Myanmar goats were categorized into Myanmar–Shan population (*n* = 5) (Yonezawa et al. 2026). All goats were genotyped using the Illumina goat SNP 50K Bead chip (53,347 SNPs) (Illumina, Inc. San Diego, California, United States).

### Dataset Construction and Quality Control

We constructed two datasets (Dataset1 and Dataset2) for use in calculating genetic diversity and other analyses, respectively. We combined our data with the previously published goat 50K SNP datasets (Colli et al. 2018; Berihulay et al. 2019; Deniskova et al. 2021; Chokoe et al. 2022; Yonezawa et al. 2026), but without the cosmopolitan Angora, commercial breeds in South Africa (Kalahari Reds and Savanna), the New World, and the Oceanian populations. This resulted in genotypes of 4,480 goats from 44 countries in the Old World and seven wild bezoars from Iran for 44,002 SNPs (Fig. S8). After merging, quality control was performed using PLINK v.1.9. SNPs with call rate <0.98 and MAF <0.01 were excluded, thus removing individuals with call rates <0.98 and LD pruning (plink indep-pairwise: window size 50 Kb, step size 5, *r*^2^ threshold 0.04). This procedure resulted in 21,009 SNPs with 4,318 individuals representing 54 wild or domestic populations from 44 countries for the analysis. We defined this dataset as “Dataset1”. For “Dataset2,” populations with >40 goats, except the population of ISEA, were randomly sampled to 40 individuals per population to reduce ascertainment bias using R script (https://github.com/RyoMasuko/ISEA_50K_Scripts). “Dataset2” consisted of 21,009 SNPs with 1,747 goats/bezoar (Table S5).

### Genetic Diversity and Population Structure Analysis

Genetic diversity was calculated at both country and subpopulation levels, while the correlation between geographic distance and expected heterozygosity focused on country-level diversity. Observed (*H*_o_) and expected (*H*_e_) heterozygosities for each SNP were calculated using PLINK 1.9 (Purcell et al. 2007). The most probable domestication center for goats is Ganj Dareh (34°16′19.56′′N, 47°28′32.88′′ E) in West Zagros of Iran and Nevali Cori (37°31′06.0′′ N, 38°36′20.0′′ E) in Taurus Mountains of Turkey (Peters et al. 1999; Zeder and Hesse 2000; Luikart et al. 2001; Daly et al. 2021). The latitude and longitude coordinates of the capital of each population were obtained from Google Maps. We calculated the average distance to Ganj Dareh and Nevali Cori from the capital of each population using distHaversine function in the R geosphere package (Hijmans 2024). Then, the Pearson correlation coefficient between the distance and *H*_e_ was calculated using the linear regression function in the R stats package (Lenth 2016). We used detectRUNS package (Biscarini et al. 2019) in R to identify ROHs and to estimate genomic inbreeding coefficient (*F*_ROH_) with the parameters: maxOppRun = 0, maxMissRun = 0, minSNP = 15, minLengthBps = 500, and maxGap = 10^6^. VCFTOOLS was used to calculate nucleotide diversity (Pi) to assess the genetic diversity of goats. Pi in VCFTOOLS was run with the parameters: window-pi 500000 window-pi-step 100000.

Unsupervised PCA and svPCA were conducted using PLINK 1.9, and the results were plotted using R 4.2.3 (R Core Team 2023). Genetic population structure and admixture using Dataset2 were estimated using ADMIXTURE version 1.3.0 (Alexander et al. 2009) with *K* = 1 to 40. RRAA was performed to suppress the inbreeding bias by reducing the representation of inbred breeds (Yonezawa et al. 2026). Because of inbreeding bias, populations with the highest levels of inbreeding are often classified as ancestral clusters. In addition, sampling bias causes overrepresented populations at low *K*-values to be inferred as ancestral clusters. Therefore, to counteract the effects of inbreeding bias, we limited the proportion of highly inbred breeds in our analysis. To achieve this, we randomly selected six individuals from the Cambodia-M population based on admixture results, ran ADMIXTURE 100 times for such selections, and averaged the percentages of the inferred genomic structure for each individual.

### Phylogeographic Inference and Admixture Events

To evaluate the differentiation within and across breeds, pairwise weighted *F*_ST_ distances between breeds were calculated using the R package StAMPP (Pembleton et al. 2013) and visualized in NeighborNet graphs using SPLITSTREE (Huson and Bryant 2024). The admixture among populations was inferred using TREEMIX 1.13 (Pickrell and Pritchard 2012) with *m* = 0 to 15 migration edges and bezoars as the outgroup. In the Treemix analysis, we defined clusters of goat populations based on the following geographical locations and RRAA pattern: Europe, Northwest Africa, Southern Africa, Eastern Africa, Boer, West Asia, Central Asia, South Asia, East Asia, MSEA, the Philippines, and Indonesia (Table S5). The Boer is a breed established through crossbreeding South African and Asian breeds (Porter et al. 2016) and is currently bred worldwide. The results of RRAA revealed a homogeneous genetic structure across Boer goats. To distinguish the impact of Boer goats from that of African regions, Boer goats were considered a single cluster in this study. To determine the shared evolutionary history between the ISEA population and other populations, we also conducted outgroup *f*_3_ statistics and *f*_4_ statistics analyses using the R package ADMIXTOOLS 2 v.2.04 (Patterson et al. 2012; Maier et al. 2023). These statistics were computed in the forms of *f*_3_ (O; X, Y) and *f*_4_ (O, X; Y, Z), where O represents the outgroup (Bezoar), Y is the ISEA population, and X and Z are other populations. For *f*_4_ statistics, statistical significance was determined at a threshold of *P* < 3.38 × 10^−7^ by Bonferroni correction with α = 0.001/2,958, where 2,958 represents the number of independent *f*_4_ statistics conducted.

## Supplementary Material

evag177_Supplementary_Data

## Data Availability

The datasets generated and analyzed during the current study are available in the Figshare repository, (https://figshare.com/, doi number: 10.6084/m9.figshare.29220125).
