# Supplementary material for: Genome-Wide Diversity of Goats From Indonesia and the Philippines Reveals Local Ancestry With Different Levels of Cosmopolitan Admixture
Source: Genome Biol Evol. 2026 Jul 17;18(7):evag177. doi: 10.1093/gbe/evag177 (PMC13403562; doi:10.1093/gbe/evag177)
Supplement: evag177_Supplementary_Data [file evag177_supplementary_data.zip › Supplementary_Figures_final.docx]

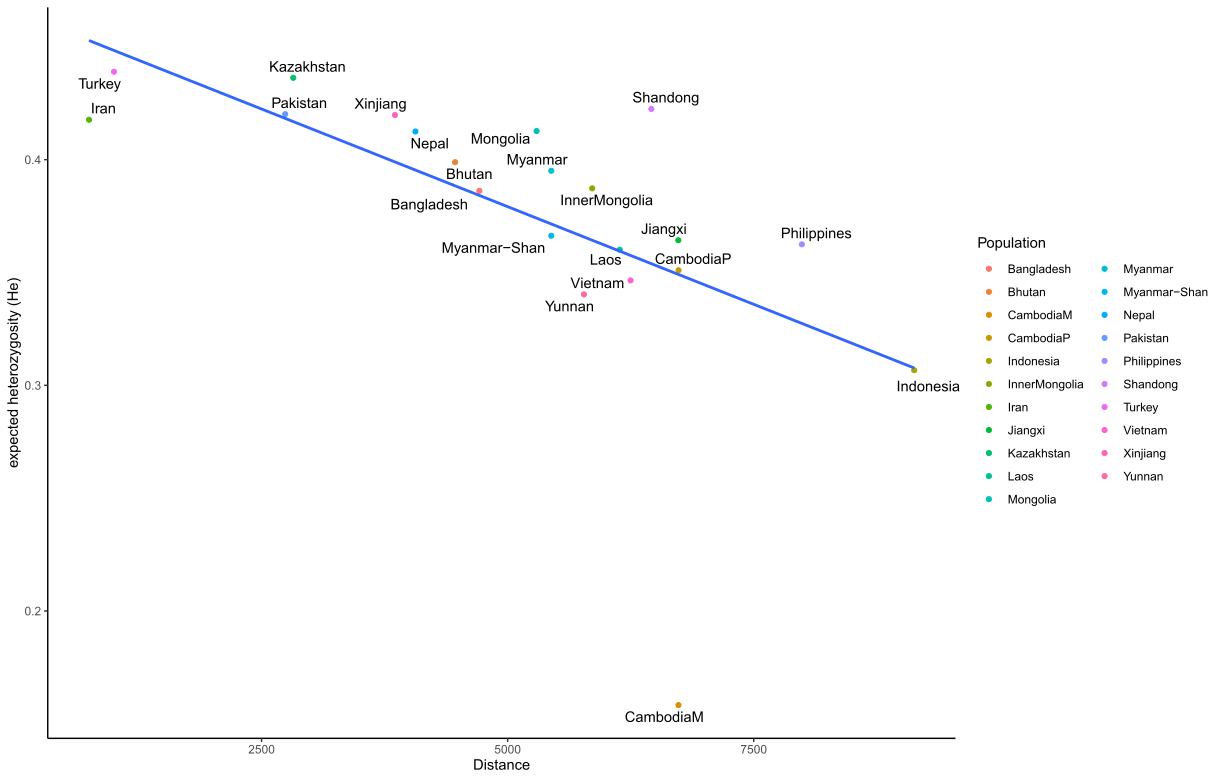


Figure S1. The correlation between expected heterozygosity of Asian goat populations and distance between their sampling locations and the domestication centers. This plot was generated using RStudio 2023.09.0+463 "Desert Sunflower".


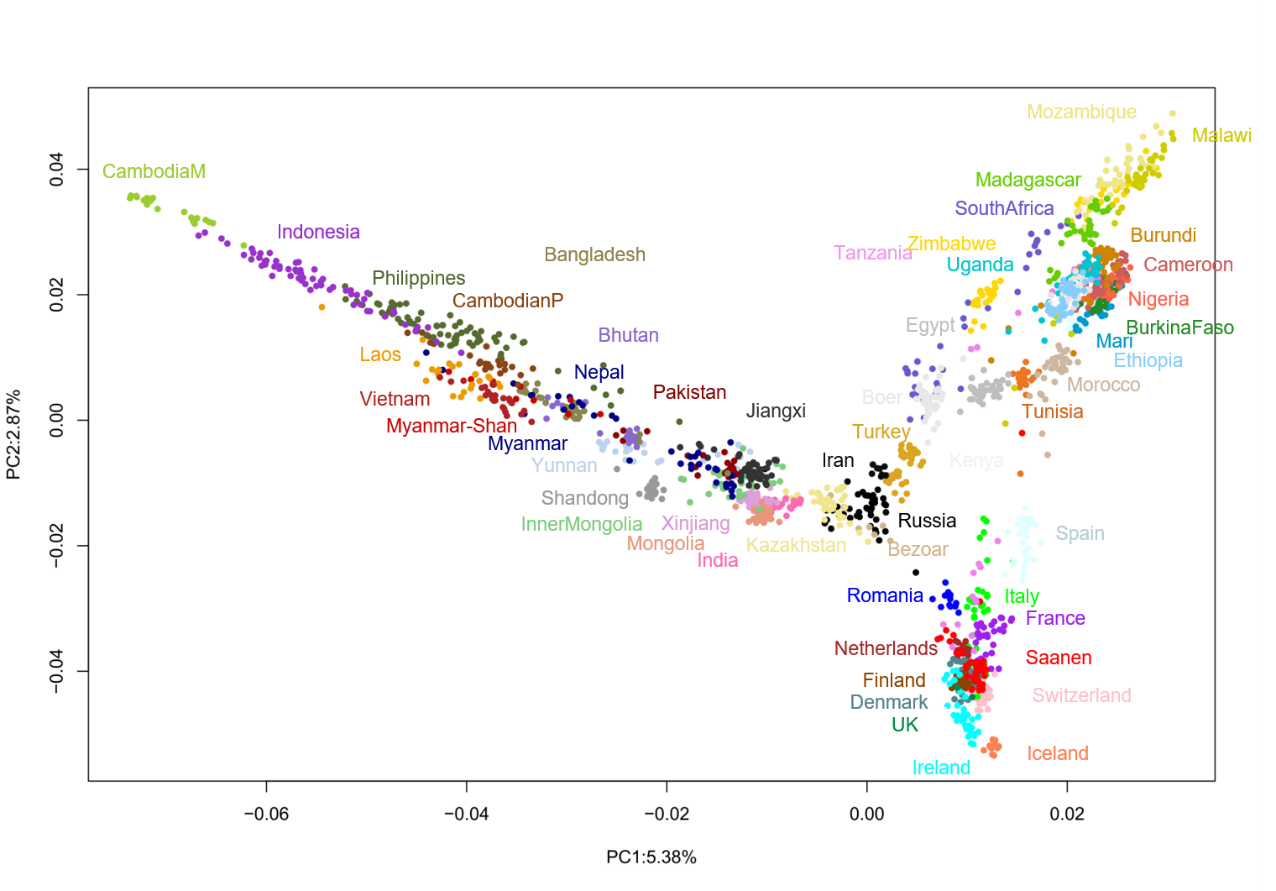


Figure S2. Unsupervised principal component analysis (PCA) on the Europe, Africa , Asian domestic goats and bezoars. This plot was generated using RStudio 2023.09.0+463 "Desert Sunflower“.


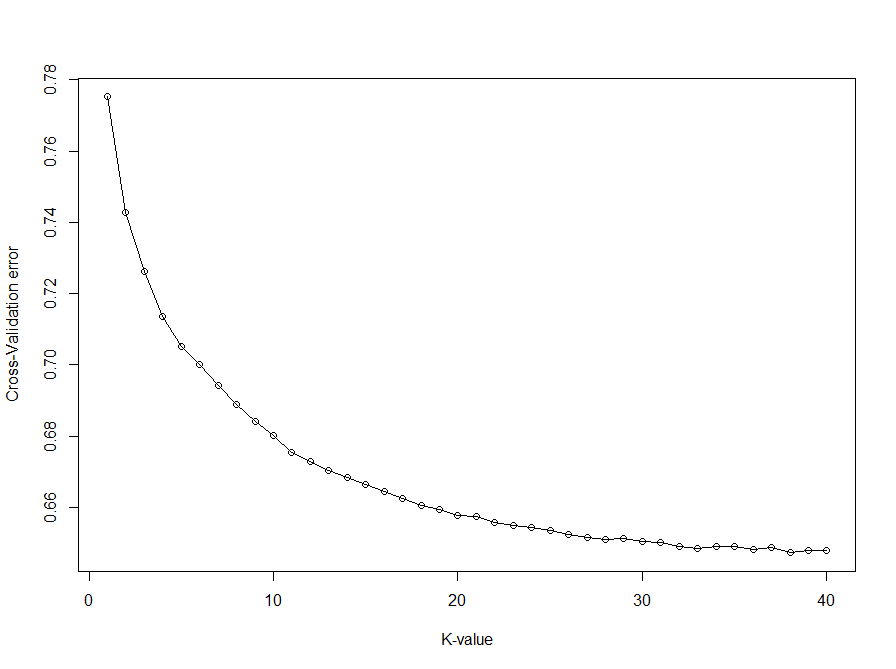


Figure S3. Plot of ADMIXTURE Cross-validation (CV) error from 1 to 40 clusters (K)


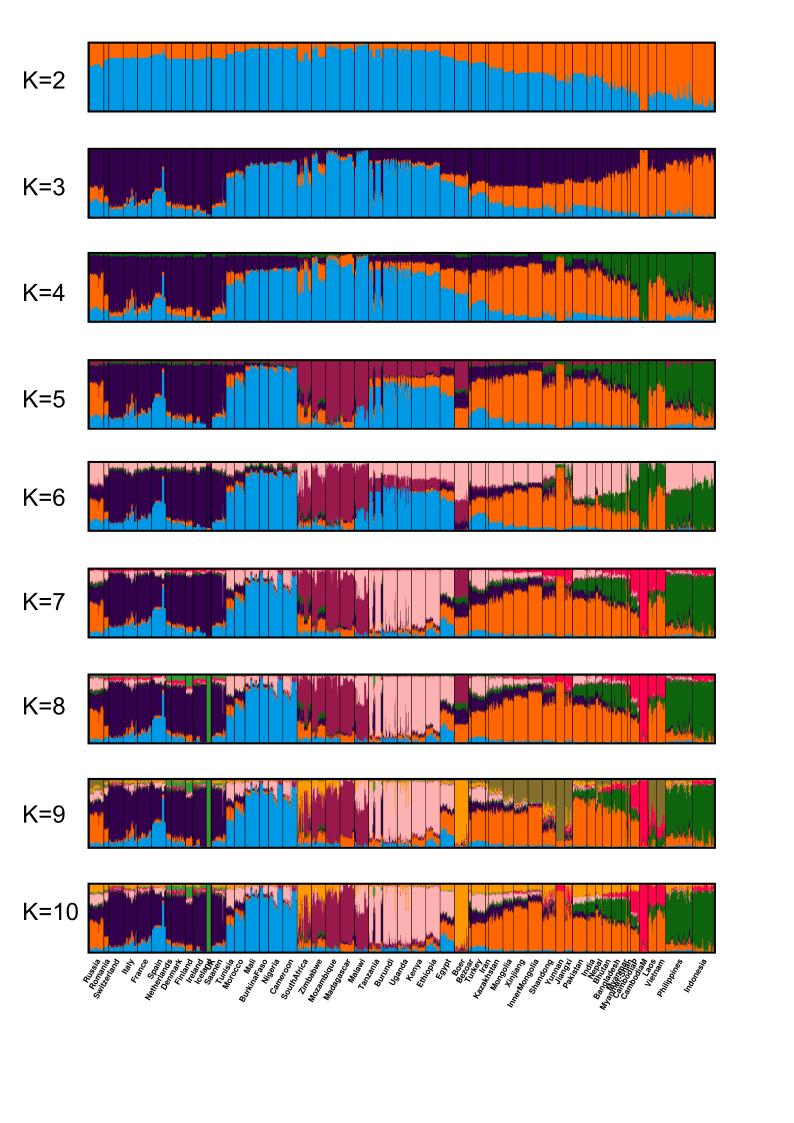


Figure S4. Admixture analysis of Eurasian and African goats at K=2 to 15, 20, 30, and 40 by the ADMIXTURE program ver. 1.3. This figure was generated using CLUMPAK (https://clumpak.tau.ac.il/).


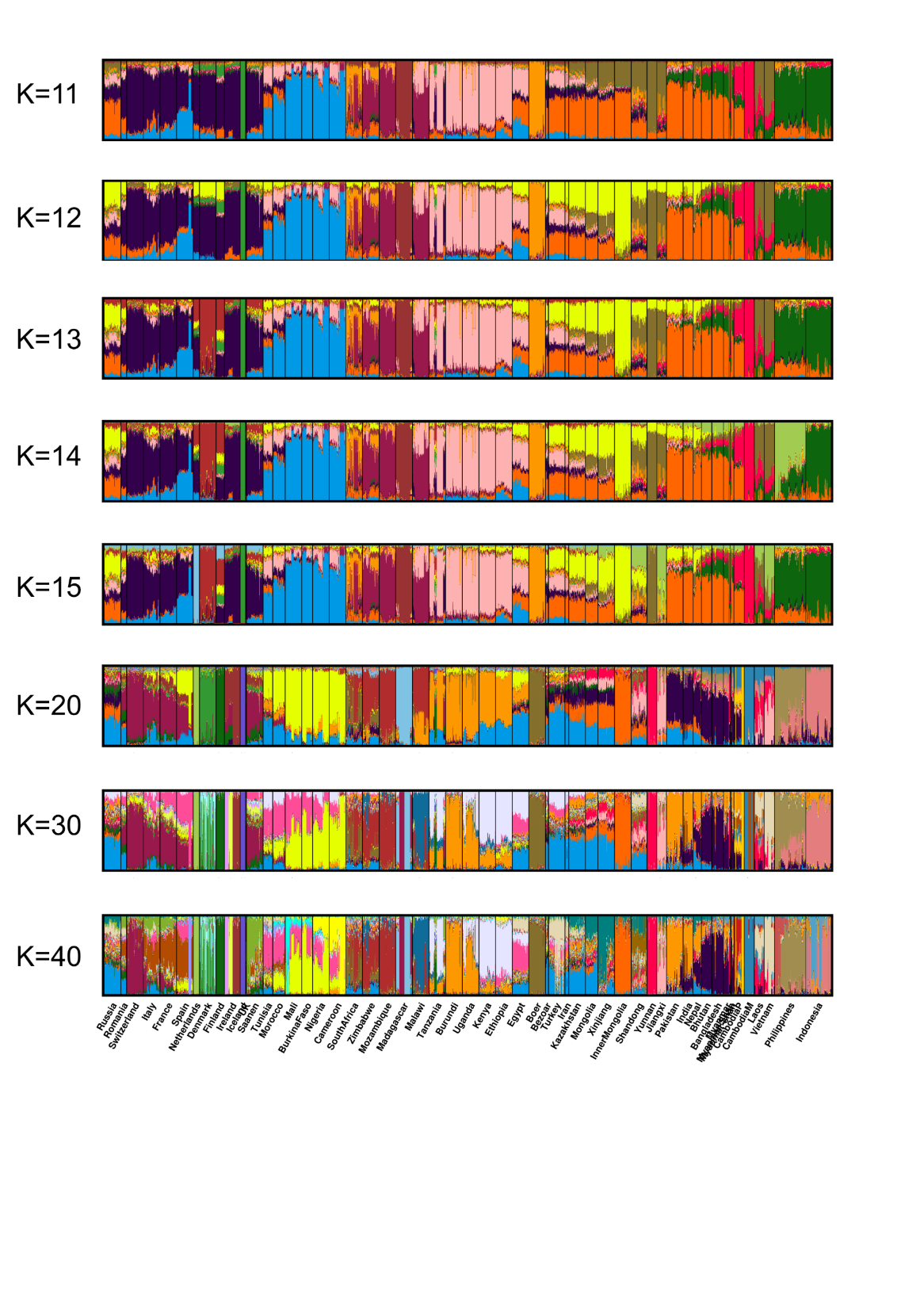


Figure S4. (continued)

Figure S5. Reduced Representation Admixture analysis (RRAA) of the Eurasian and African goats by the ADMIXTURE program ver. 1.3 (K=2-8). This figure was generated using CLUMPAK (https://clumpak.tau.ac.il/).


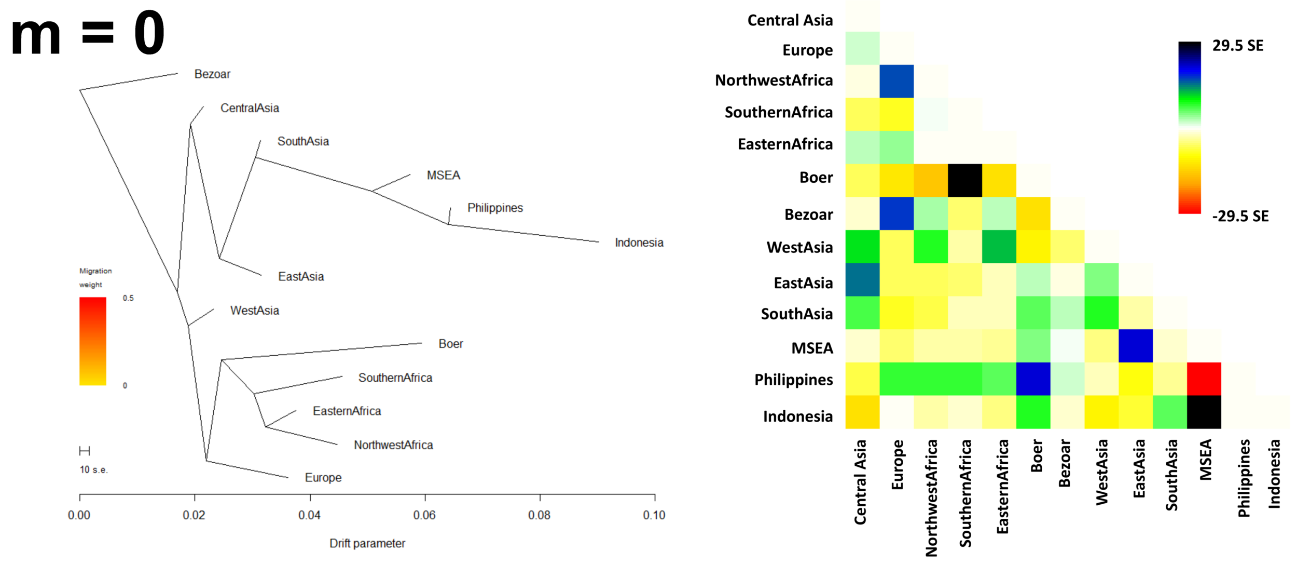

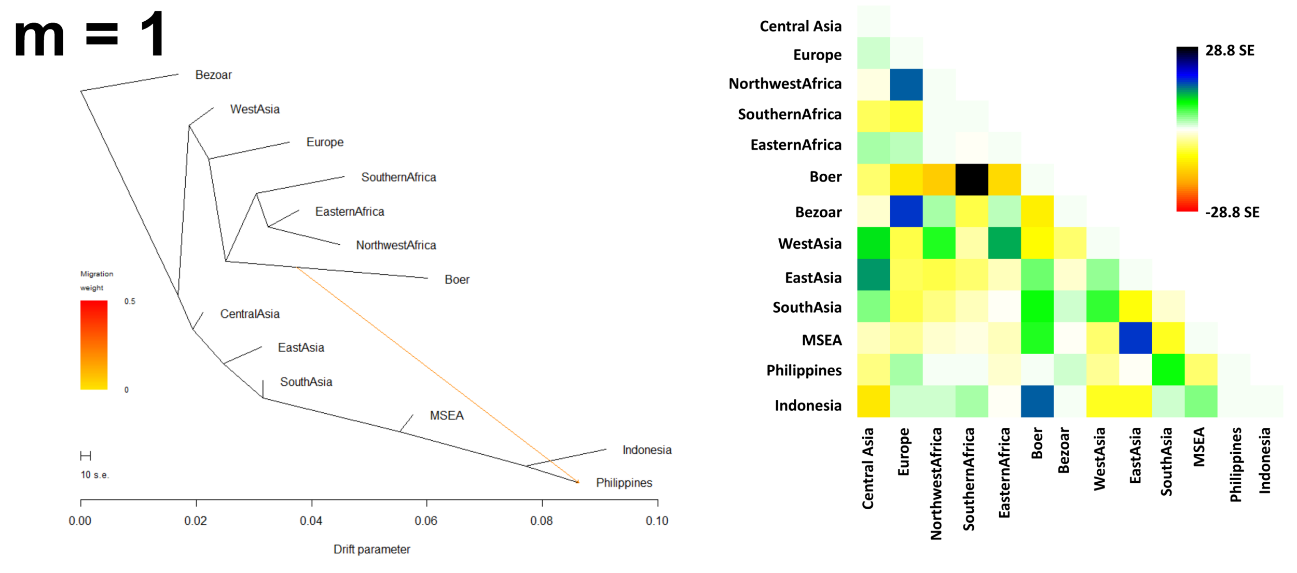

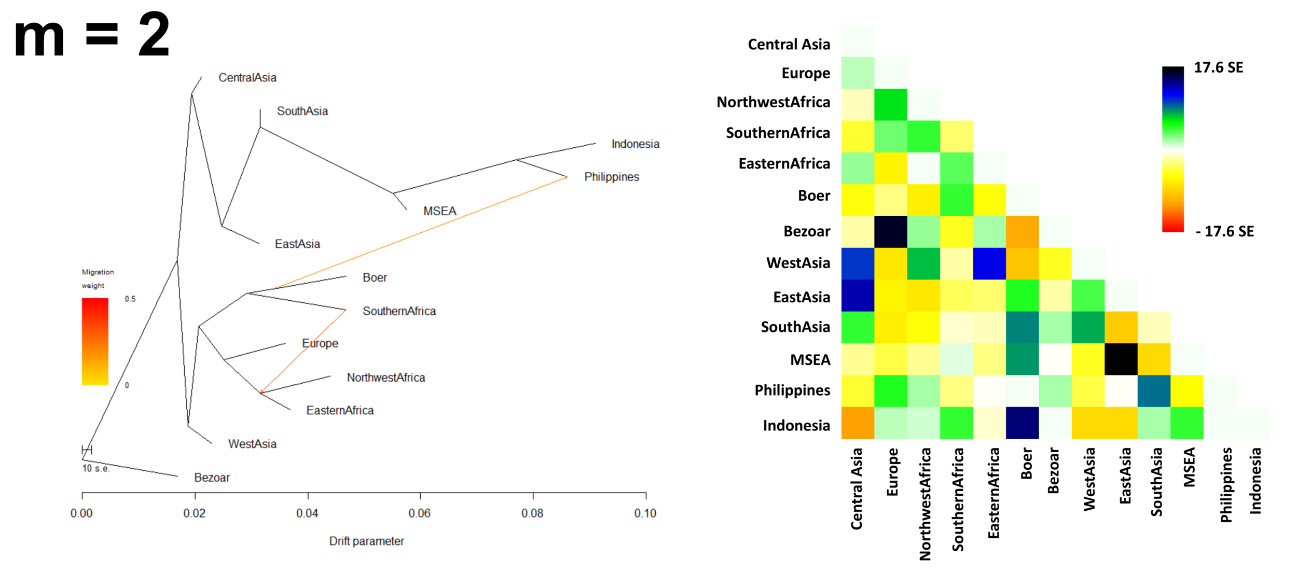


Figure S6. Regional Treemix graphs (left) and residual plot (right) of Eurasian and African goats at m=0 to 15. Migration edges are colored in accordance with their migration weights. Scale bar indicates the drift parameter. Abbreviation: MSEA, Mainland Southeast Asia.


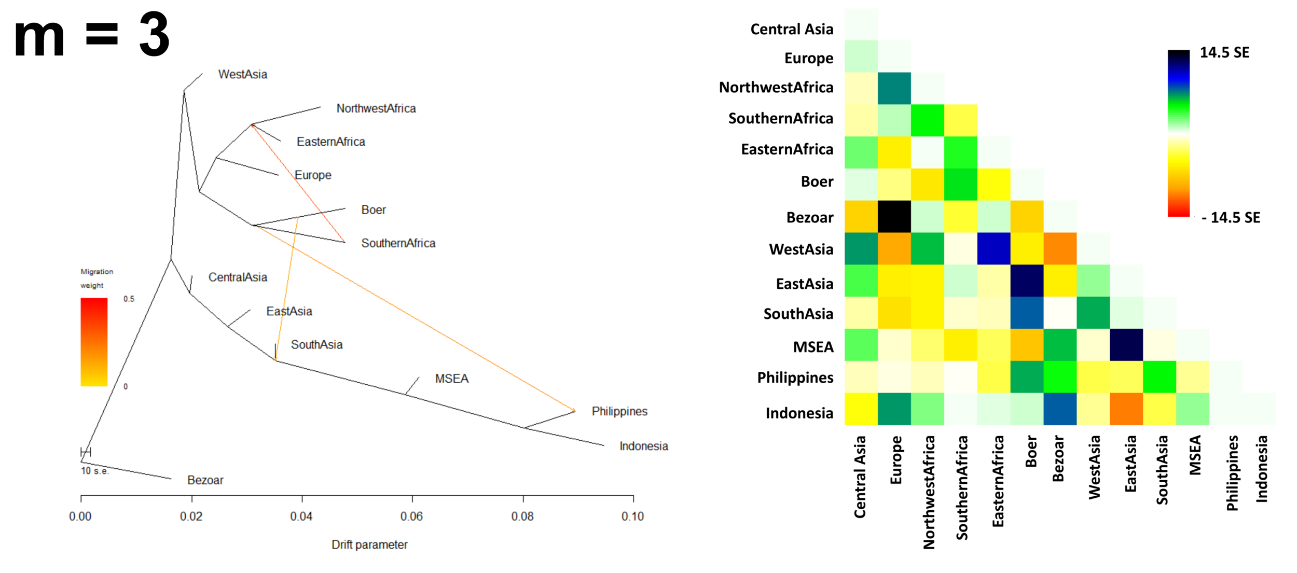

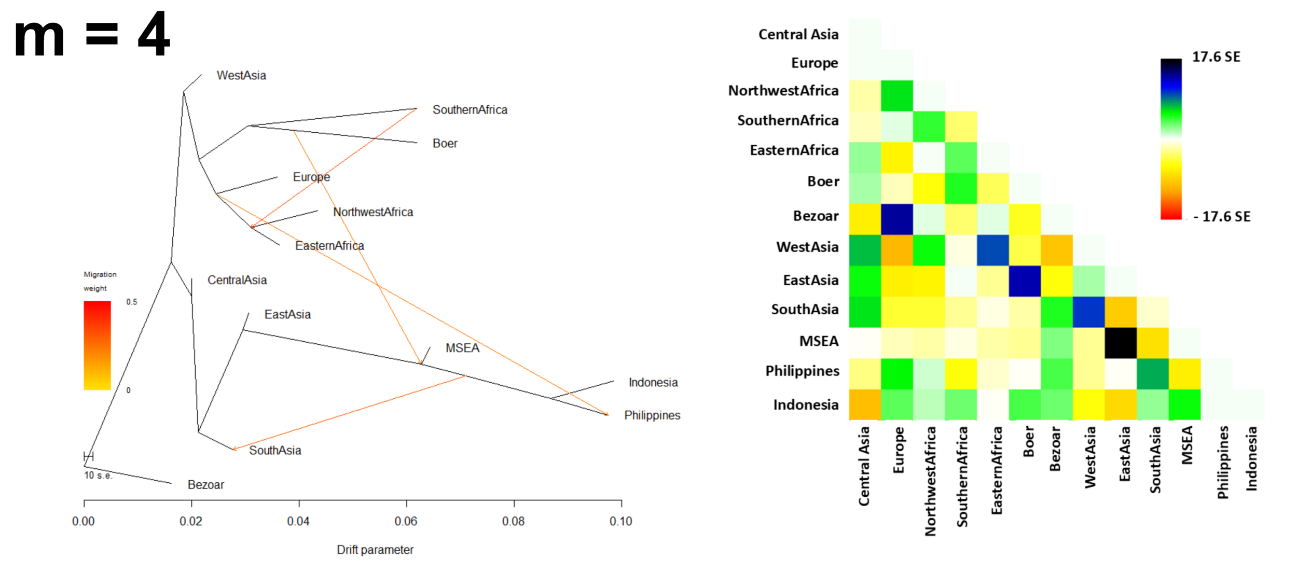

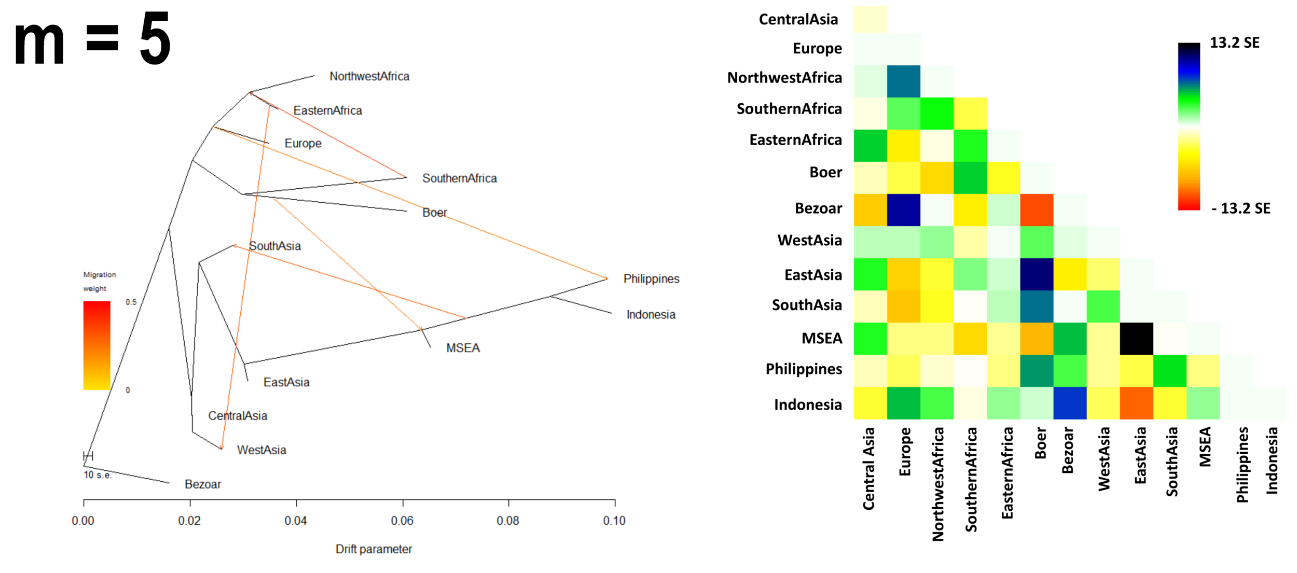


Figure S6. (continued).


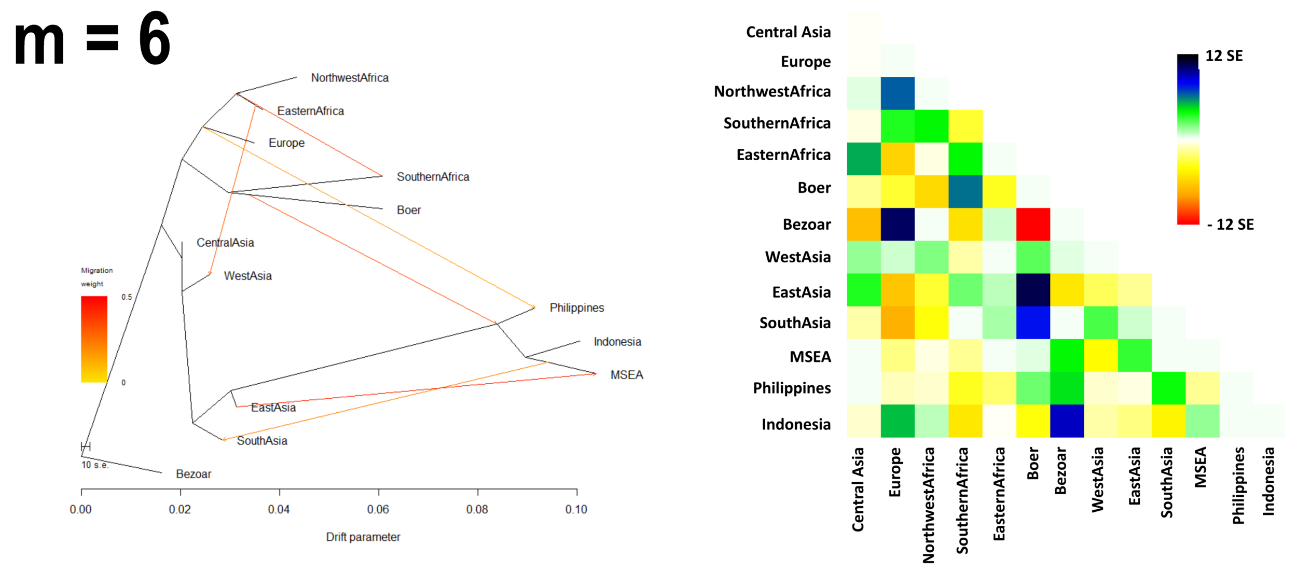

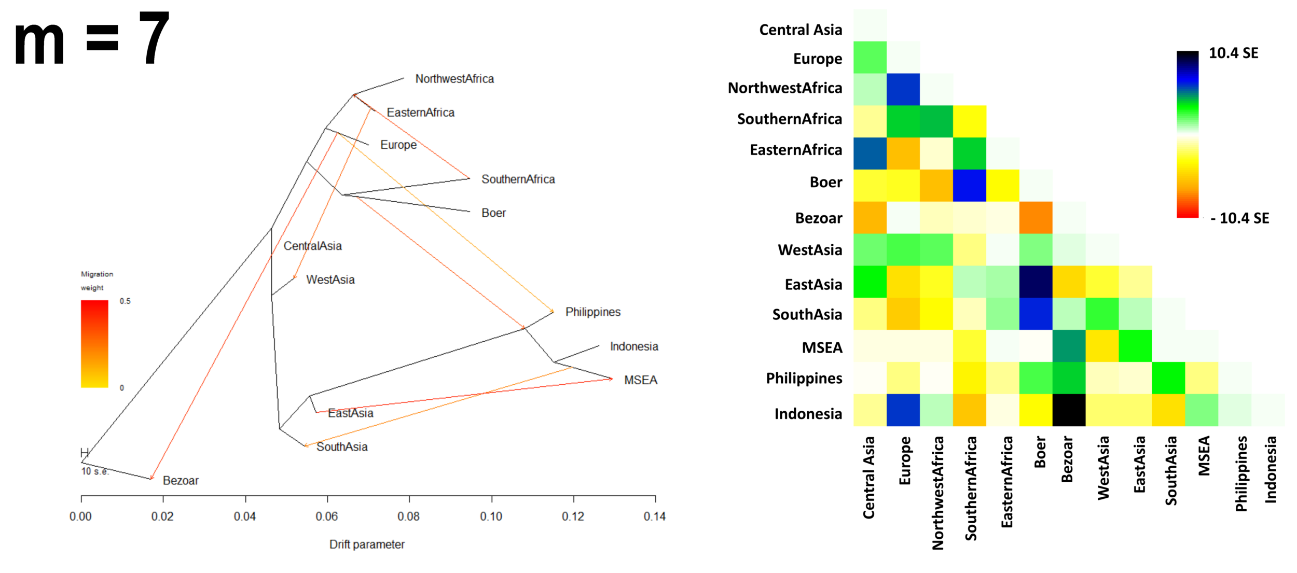

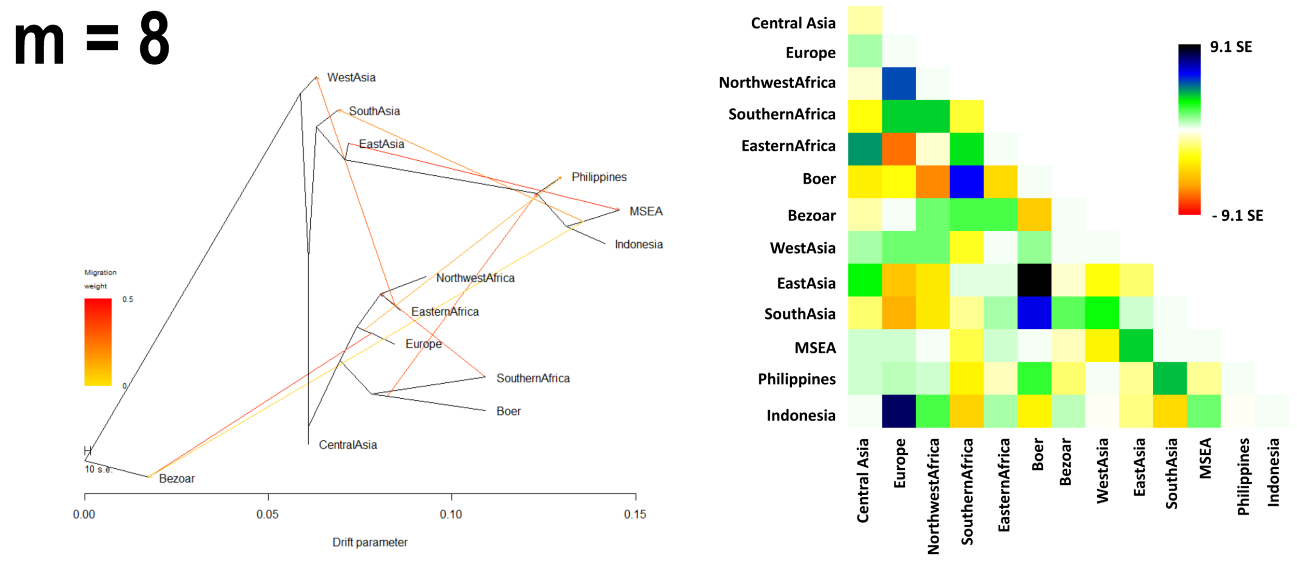


Figure S6. (continued).


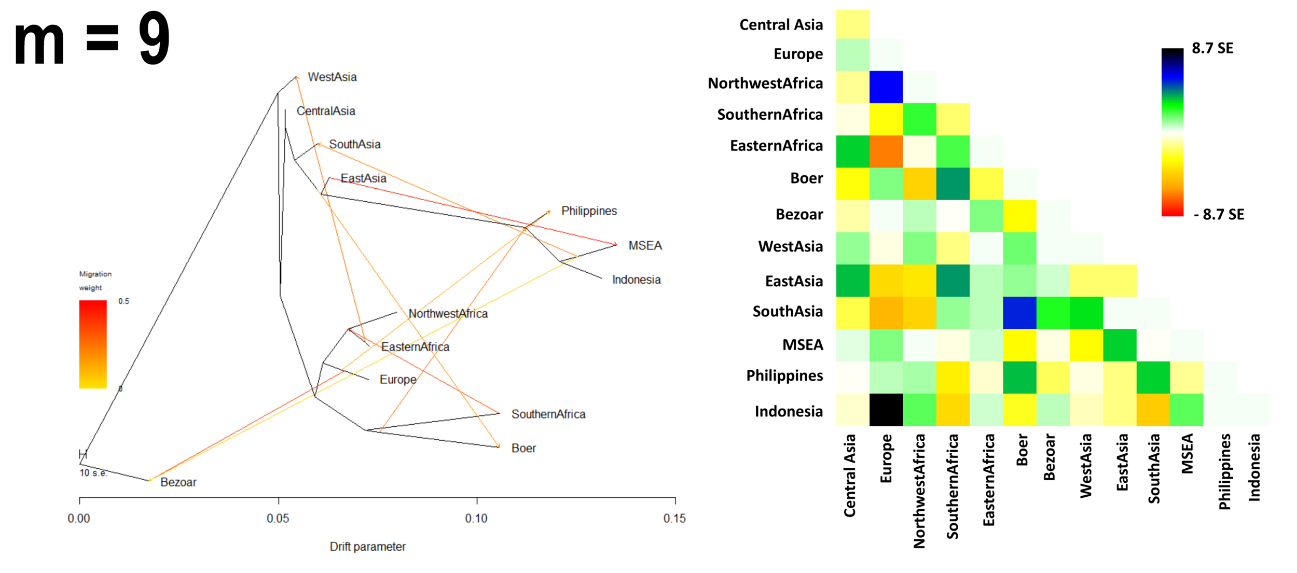

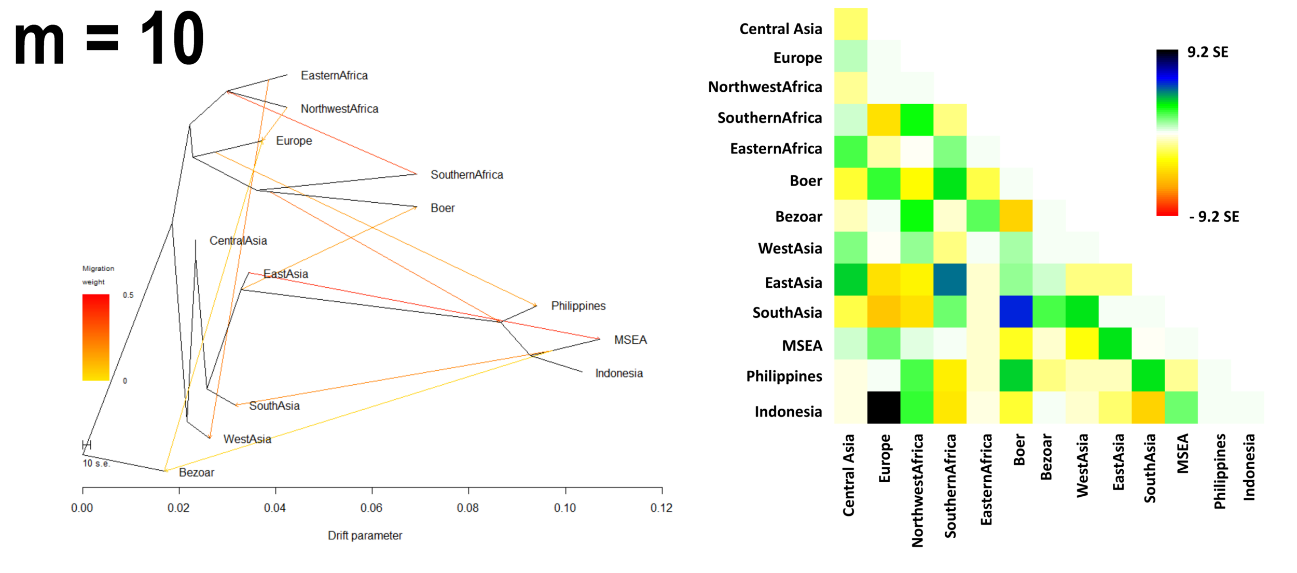

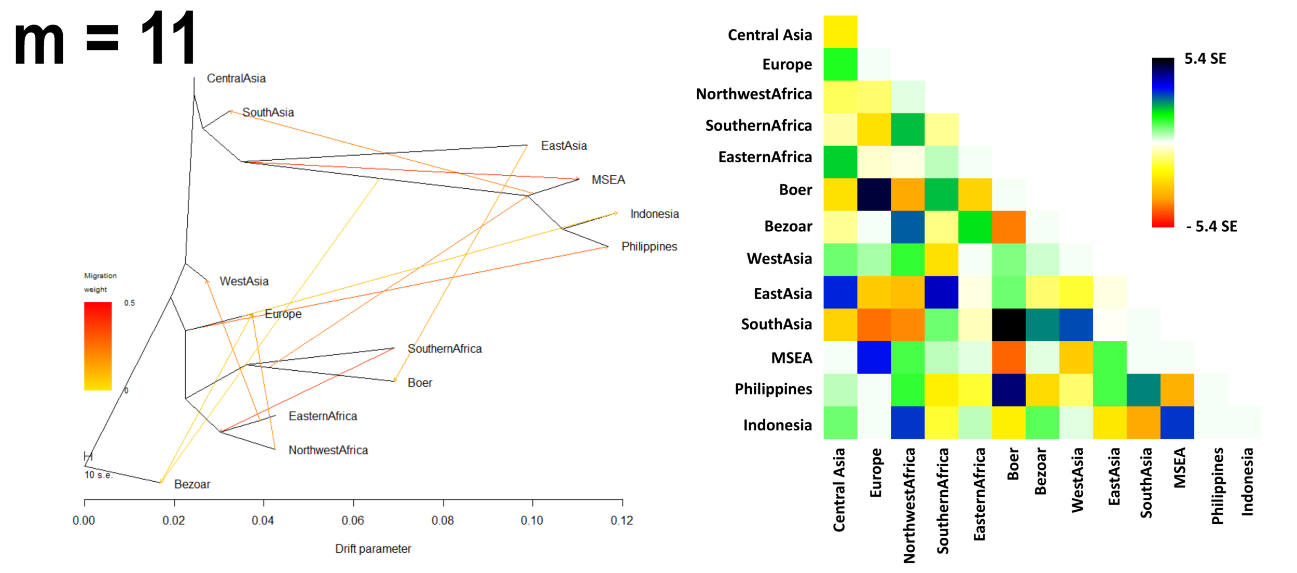


Figure S6. (continued).


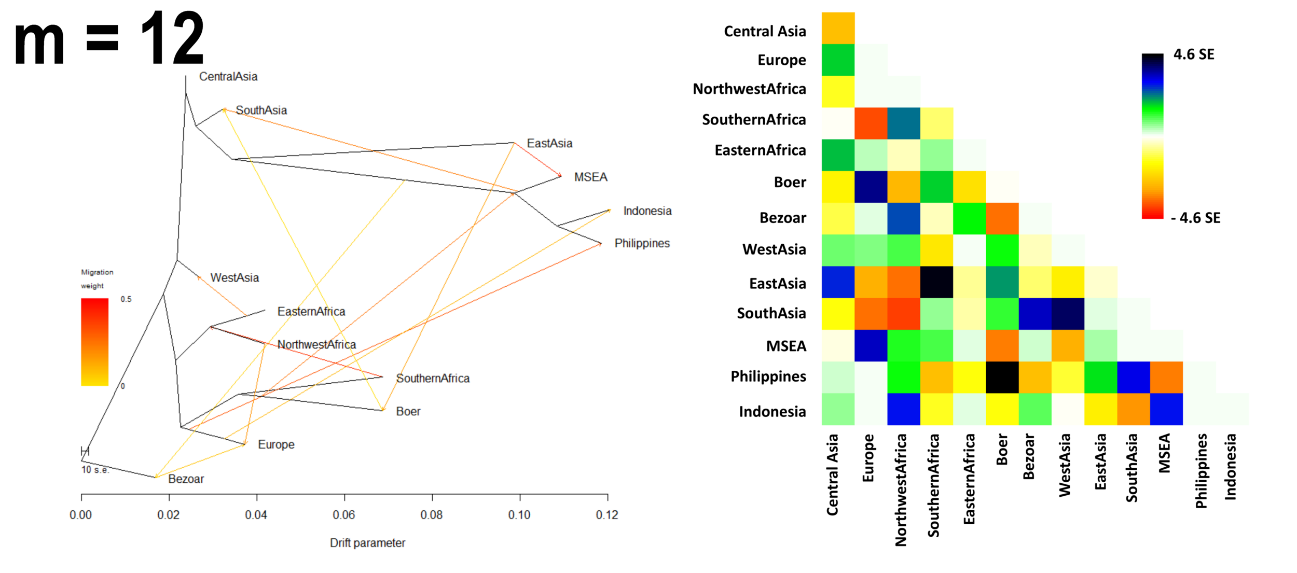

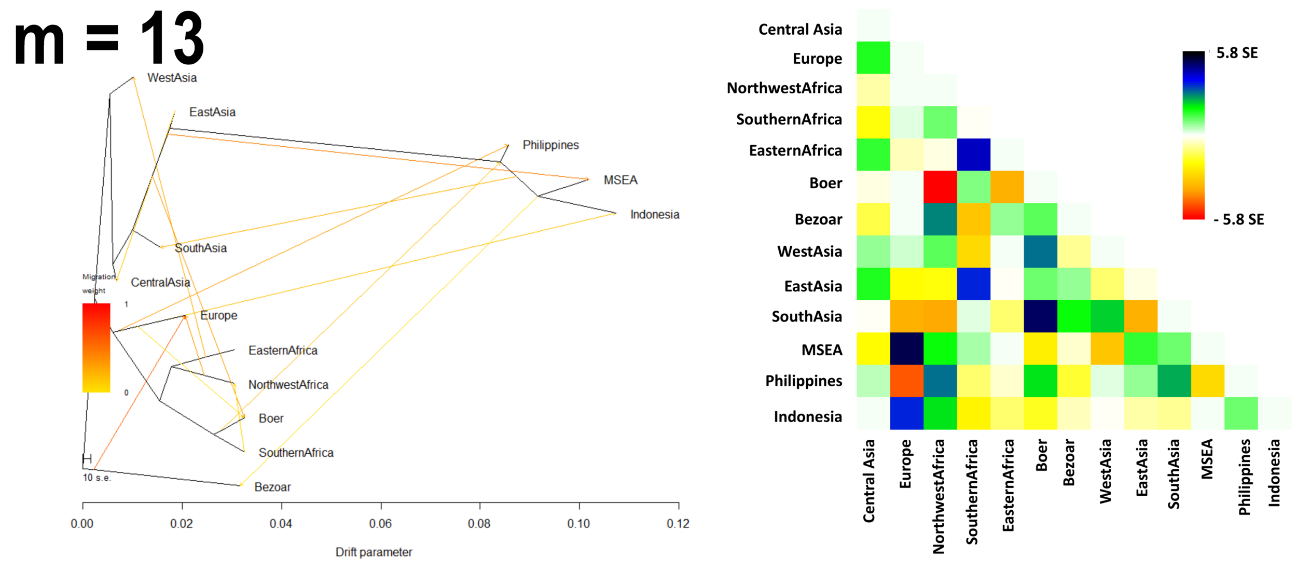

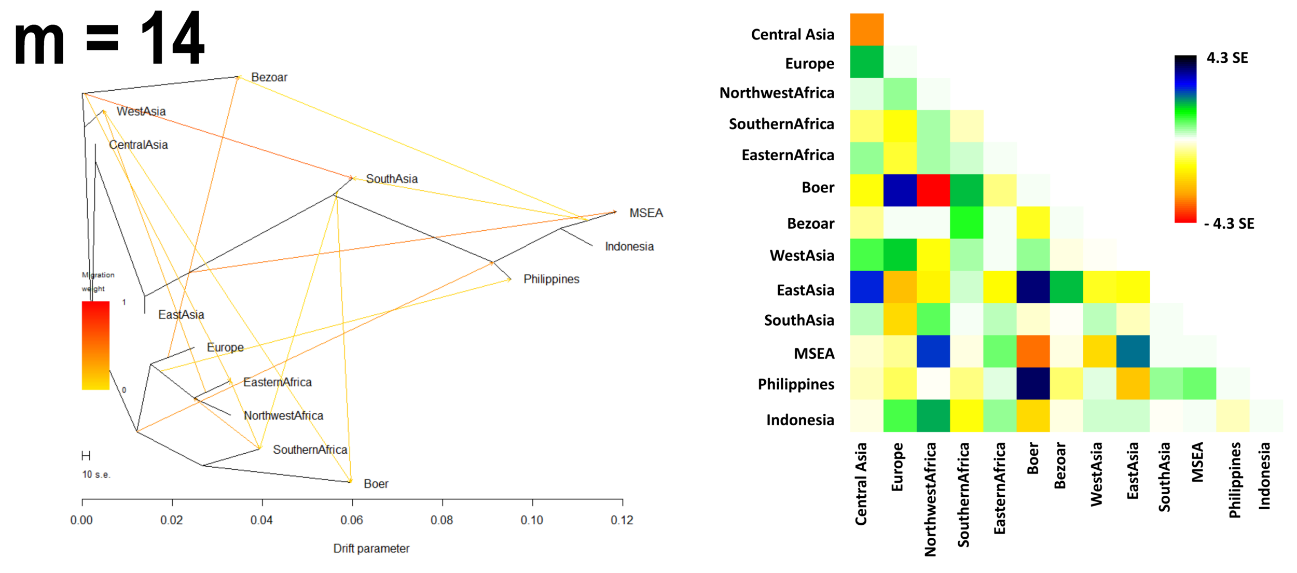


Figure S6. (continued).


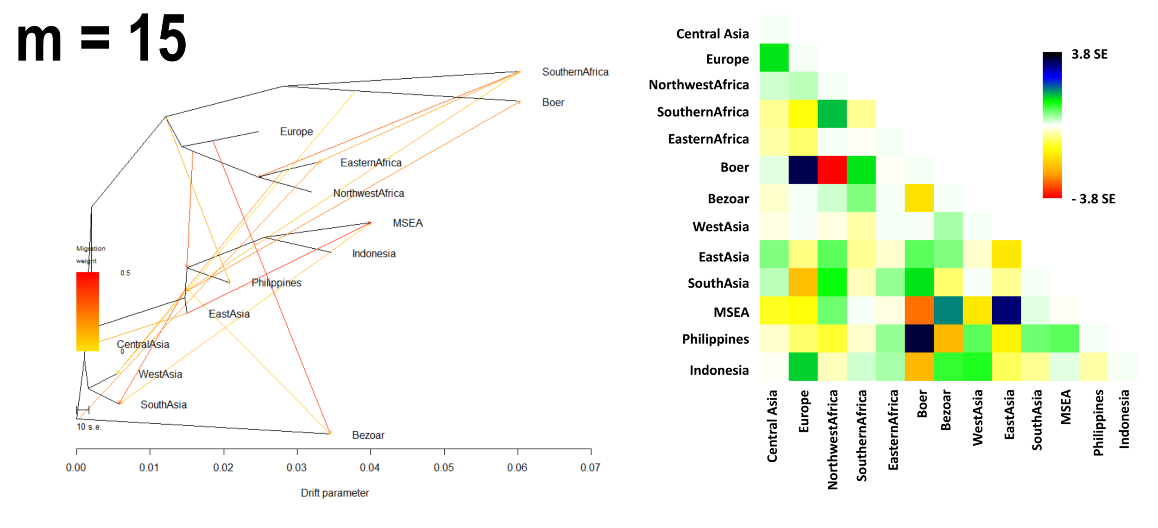


Figure S6. (continued).


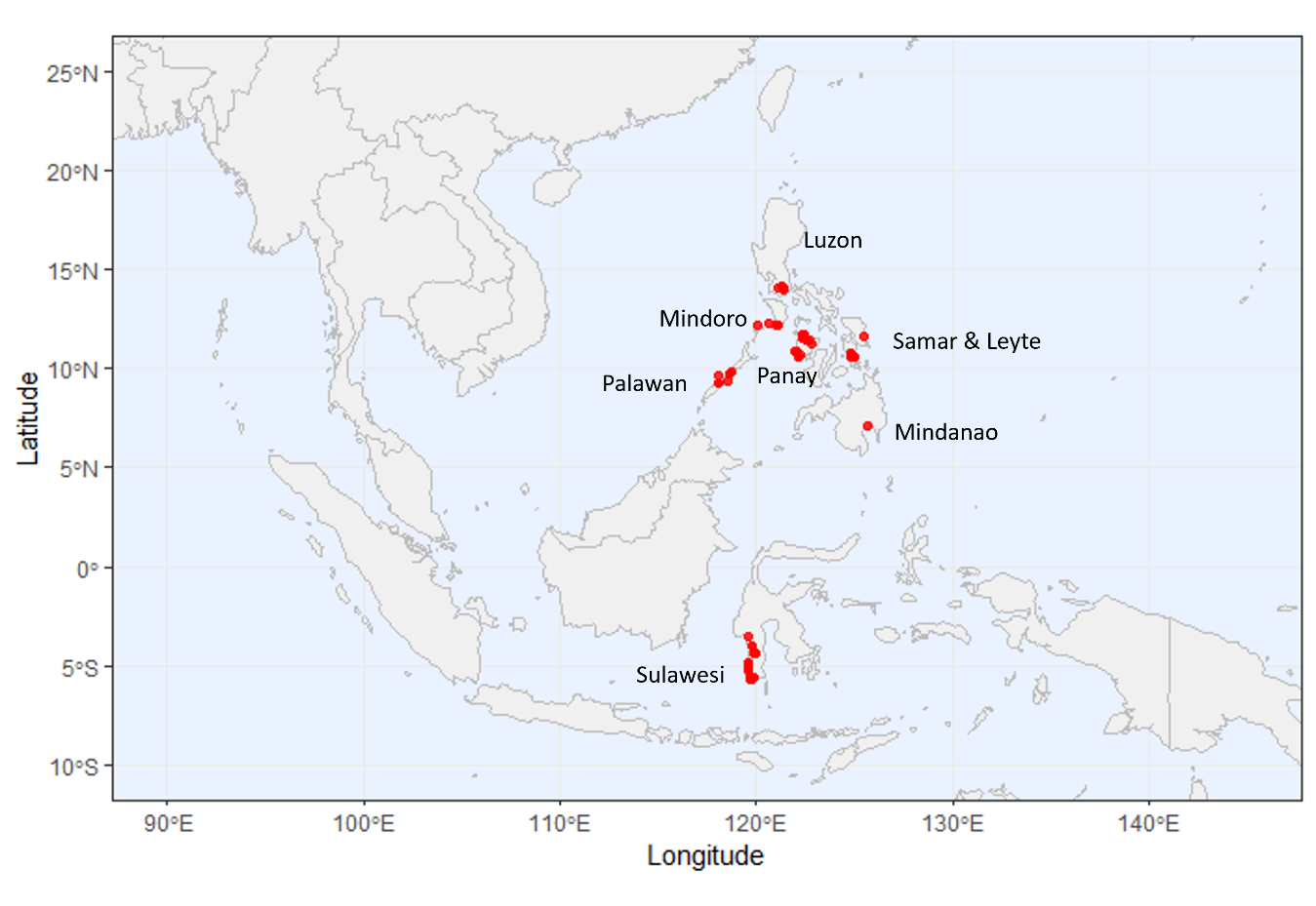


Figure S7. Sampling locations of ISEA goats in this study. Red dot indicate sampling sites. This map was generated in R studio 4.2.3 using R packages rnaturalearth and rnaturalearthdata.


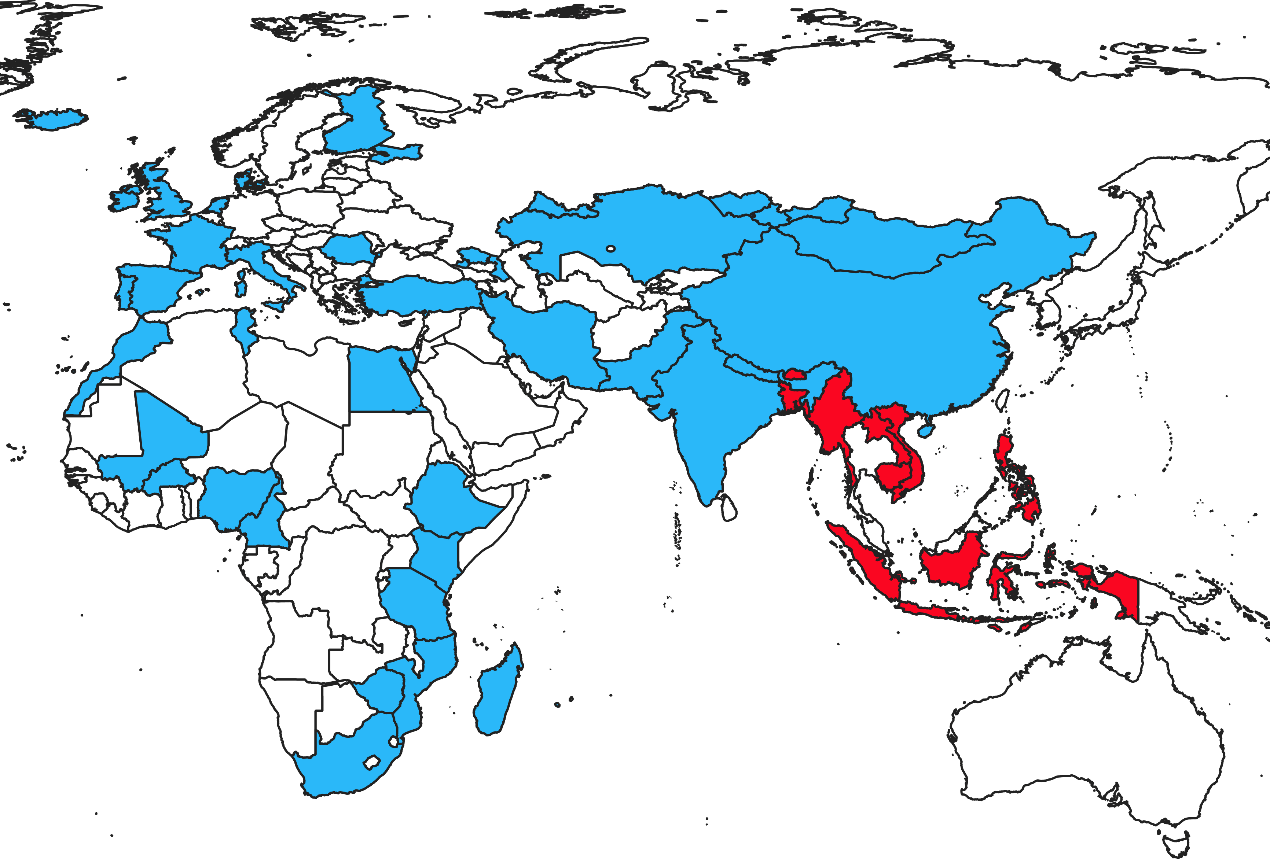
Figure S8. Geographic distribution of Old-World goats used in this study. Red and blue colors indicate the locations of goat populations used in this study and in previous studies (Colli et al., 2018; Berihulay et al., 2019; Deniskova et al., 2021; Chokoe et al., 2022; Yonezawa et al., 2026), respectively. The map was created using QGIS version 3.44.8 (https://qgis.org/) with vector and raster map data from Natural Earth (naturalearthdata.com).
